# A Robust Solar Evaporator with Hierarchical Architecture for Ultrahigh Efficiency and Large‐Scale Zero‐Liquid‐Discharge Desalination

**DOI:** 10.1002/advs.202517735

**Published:** 2025-11-07

**Authors:** Lili Sun, Ning Wang, Ning Hu, Yongyun Zhao, Libin Zhao, Wenbo Chen

**Affiliations:** ^1^ School of Mechanical Engineering Hebei University of Technology Tianjin 300401 China; ^2^ Kuojing Intelligent Manufacturing (Tianjin) High Tech Co., Ltd Tianjin 300192 China; ^3^ State Key Laboratory of Reliability and Intelligence Electrical Equipment Key Laboratory of Advanced Intelligent Protective Equipment Technology Ministry of Education and School of Mechanical Engineering Hebei University of Technology Tianjin 300401 China; ^4^ Key Laboratory of Hebei Province on Scale‐Span Intelligent Equipment Technology Hebei University of Technology Tianjin 300401 China

**Keywords:** hierarchical architecture, high evaporation performance, self‐sustainable irrigation, solar desalination, zero liquid discharge

## Abstract

Solar‐driven desalination offers a green and sustainable strategy for solving the issue of freshwater scarcity. However, it remains a challenge to develop a robust evaporator via an eco‐friendly strategy, capable of resolving the long‐standing trade‐off between the water supply, salt accumulation, and thermal localization. Herein, a hierarchical solar‐absorbing architecture derived from mycelium is developed for high‐performance desalination. With the merits of multi‐level spatially low‐tortuosity channels, gradient wettability and directional transport, the evaporator achieves an impressive evaporation rate (up to 10.25 kg m^−2^ h^−1^ for 25 wt.% brine), efficient salt collection (1.95 kg m^−2^ h^−1^ for 25 wt.% brine), zero liquid discharge desalination, and long‐term stability (over 145 h in 10–25 wt.% brine). Moreover, the scaled‐up desalination system exhibits an ultrahigh freshwater production rate of 6 kg m^−2^ h^−1^ and a daily freshwater yield of up to 32 L m^−2^ during the outdoor desalination test. A continuous desalination‐cultivation platform demonstrates the feasibility of the evaporator for self‐sustainable agricultural irrigation. This low‐cost, mycelium‐based hierarchical architecture opens an avenue to advance green and sustainable strategy for highly efficient and large‐scale desalination of high‐salinity brine.

## Graphical abstract

This study presents a low‐carbon footprint, robust 3D (three‐dimensional) evaporator featuring multi‐level low‐tortuosity channels for highly efficient desalination. It achieves an outstanding average evaporation rate of 8.1 kg m^−2^ h^−1^, salt collection rate of 1.95 kg m^−2^ h^−1^, and durability in high‐salinity brine. It also exhibits excellent outdoor water production rate, enabling a self‐sustainable agricultural irrigation for crop growth.

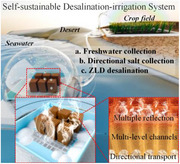

## 1 Introduction

Leveraging solar energy's inherent abundance and sustainability, solar‐driven desalination emerges as a highly promising solution to critical global challenges encompassing water scarcity, insufficient clean energy access, and carbon neutrality goals.^[^ ^1^, ^2^ ^]^ Critically, interfacial solar steam generation circumvents the negative environmental impacts associated with traditional desalination technologies, including their high energy consumption, complex treatment processes, significant costs, and risks of secondary pollution from fuel combustion or discarded membranes.^[^ ^3^, ^4^, ^5^, ^6^ ^]^ The past few years have witnessed great advances in boosting the solar evaporation rates for the interface photothermal evaporator,^[^ ^2^, ^7^ ^]^ commonly by enhancing the light absorption capacity of photothermal materials,^[^ ^8^, ^9^ ^]^ introducing microstructures and surface patterns,^[^ ^10^, ^11^, ^12^ ^]^ incorporating cold evaporation surfaces in 3D (three‐dimensional) evaporators.^[^ ^13^, ^14^, ^15^ ^]^ However, the simultaneous integration of excellent solar‐thermal conversion, thermal management, salt resistance, and durability in solar evaporators remains a significant challenge. The mismatch between the evaporation rate at the heating interface and the redissolution rate of salt will lead to either the accumulation of salts in the photothermal layer, clogging the evaporation channel, or accelerating heat loss, both of them will result in dramatically decreased evaporation efficiency, especially for high‐salinity seawater.^[^ ^16^, ^17^, ^18^ ^]^ Therefore, the conflict between preventing salt accumulation and minimizing heat loss remains the most challenging bottleneck that severely obstructs solar interfacial evaporation toward long‐term stability evaporation for practical applications.

To address salt accumulation, various strategies have been developed, including Janus evaporation surface design,^[^ ^19^, ^20^, ^21^ ^]^ vertically aligned channels design for salt circulation,^[^ ^8^, ^11^, ^22^, ^23^ ^]^ and salt deposition at specific sites.^[^ ^24^, ^25^, ^26^ ^]^ The Janus evaporators, leveraging its unique asymmetric wettability configuration,^[^ ^27^ ^]^ demonstrate remarkable advantages in solar interfacial evaporation, including efficient salt rejection and thermal localization.^[^ ^28^ ^]^ Nevertheless, this strategy faces unresolved challenges of material stability and interfacial bonding strength in environmental stability and durability for practical applications. Most importantly, the trade‐off effect between water supply and thermal localization has severely restricted the evaporation efficiency for most Janus evaporators. Optimizing the water transport paths is essential to minimize thermal losses during transport, yet it presents a dual challenge.^[^ ^1^, ^17^ ^]^ While submicron pores enhance thermal localization, they restrict salt‐ion diffusion, triggering crystallization. Conversely, macropores achieve exceptional water transport, yet their propensity to exacerbate energy losses due to thermal conduction and convection remains a critical barrier. Another solution is to construct the vertically aligned water channels for shortening the water transport path and promoting the salt cycle.^[^ ^29^ ^]^ However, the adoption of back diffusion and convection leads to significant conduction heat loss to the bulk water, reducing evaporation efficiency. Moreover, the resulting concentrated brine is discharged into nearby water bodies, causing severe environmental harm. The salt crystallization strategy offers an effective solution for zero liquid discharge (ZLD) desalination,^[^ ^30^, ^31^, ^32^ ^]^ yet most of the current evaporators suffer from low mechanical strength, the regular removal of salt crystals will inevitably damage the structure of the material and lead to poor durability and stability of solar evaporators. In addition, many efficient solar evaporators usually require hazardous or time‐consuming preparation processes, such as toxic reagents,^[^ ^33^ ^]^ high‐pressure or high‐thermal treatments.^[^ ^34^ ^]^ Therefore, it remains formidable to develop a robust evaporator via an eco‐friendly strategy, capable of mitigating liquid reflux and convective energy losses, while resolving the trade‐off between the water supply and thermal localization, so as to achieve high‐efficiency, long‐term stability, and ZLD desalination of hyper‐saline brine (> 15 wt.%).

The advancements in evaporators depend on the synergies among multiple critical issues, it is crucial to engineering a hierarchically integrated structure and functions for practical applications. Unfortunately, current researches predominantly focus on optimizing one or a few critical factors due to the constraint of conventional manufacturing processes and raw materials. Biological systems evolve hierarchical architectures with adaptive environmental response.^[^ ^35^ ^]^ The synergistic structure‐chemistry coupling in their cross‐scale assemblies defines an evolutionary refined paradigm for advanced material design. *Bracket fungi*,^[^ ^36^, ^37^ ^]^ a genus of *Basidiomycota*, have received great attention in the field of Chinese medicine for thousands of years, yet their hierarchical structure and material behavior have long been neglected. *Bracket fungi* exhibit remarkable tolerance to adverse climate conditions, including torrential rain scouring, prolonged drought stress, extreme temperature fluctuations, alongside biotic stresses from pathogens. Its unique environmental resilience stems from the delicate synergistic coupling of hierarchical structures and surface chemical moieties: i) The macroscopic layered architecture favors the diversion of the rainwater, while the surface hydrophobic structure enforces infiltration resistance; ii) Its drought adaptation relies on the functionally graded pores for moisture holding and the embedded hydrophilic moieties for water molecular retention. iii) Hierarchical foam‐like structure of nanofibers enables humid‐heat or frigid extremes resilience through multiscale thermal management mechanism, including physical barrier, adaptive thermal regulation, and protective molecular shields. Therefore, the *Bracket fungi* possess adaptive water transport and remarkable thermal management, and are expected to be a promising material for solar‐driven interfacial evaporation.

**Figure 1:**
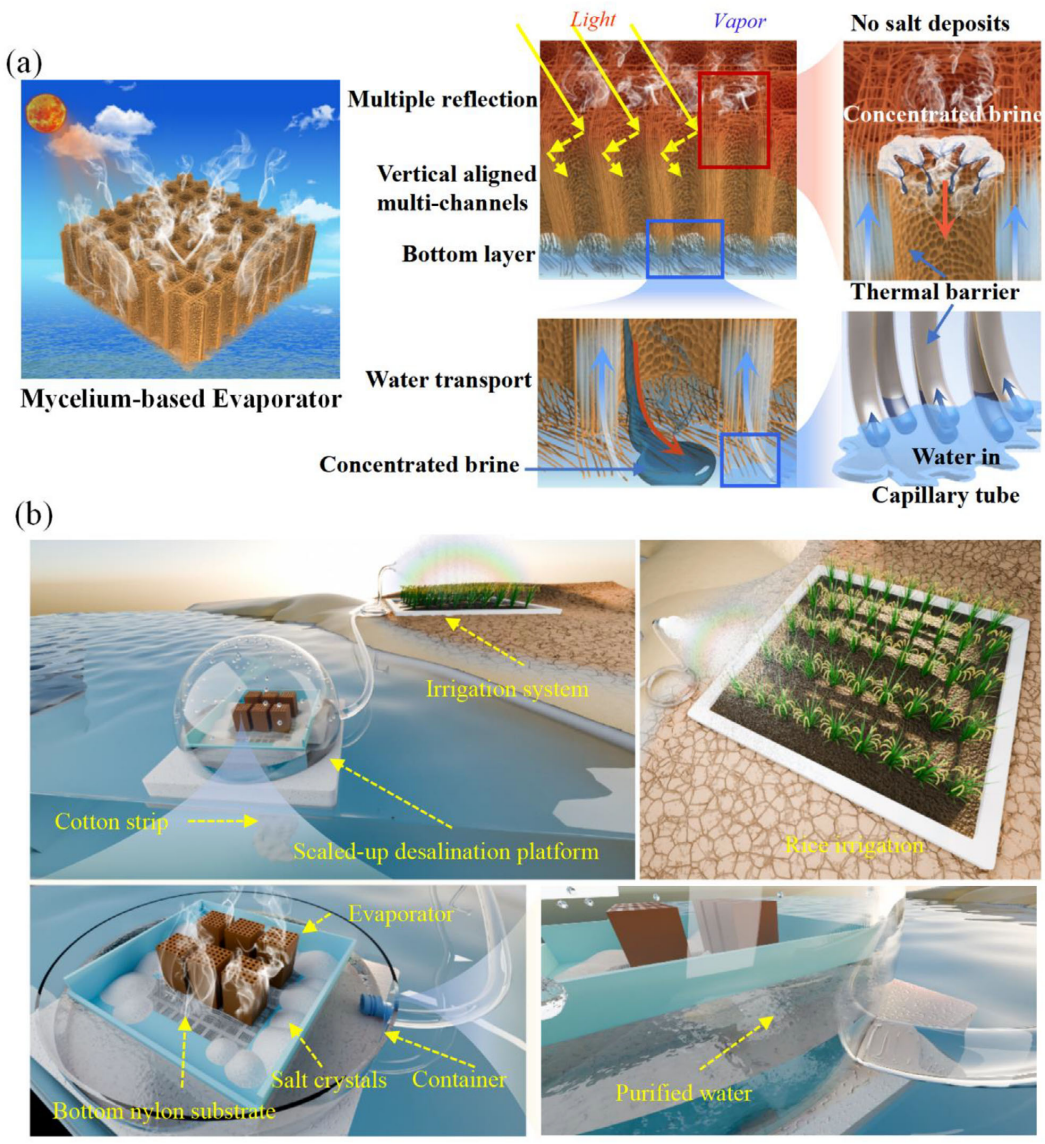
Schematic illustration of the mycelium‐based solar evaporator and its practical application. a) Diagram of the hierarchical evaporator and its solar evaporation mechanism. b) Schematic diagram of the continuous desalination‐irrigation system for freshwater production and crop growth.

Herein, we propose a simple and eco‐friendly strategy to build high high‐performance mycelium‐based interface solar evaporator by using the native fruit bodies of *Bracket fungi*. As shown in **Figure** **1**a, the mycelium‐based evaporator comprised a 3D hyphae skeleton featuring ordered hierarchical structure. The hierarchical structure exhibits vertically arranged channel arrays and the bottom horizontal spongy layer. Specifically, 3D vertical hyphae skeleton integrates triple spatially low‐tortuosity channels: i) The nano‐sized capillary structure of the hyphae promoted the water transport (Figure S2, Supporting Information). Thanks to the excellent water transport property, the evaporator can obtain a large effective exposure height, contributing to an enhanced environmental energy harvesting; ii) The hyphae skeleton acting in conjunction with its gradient wettability effectively resolve the trade‐off between the water supply, salt accumulation and thermal localization; iii) The vertical aligned micro‐sized channels enable the concentrated brine to re‐flux (Figure S3, Supporting Information), while the evaporator's side can absorb heat from the surrounding environment through convection and radiation, which can not only improve the evaporation efficiency, but also effectively compensate for the heat loss of the top surface. The bottom layer also favors the transportation of the concentrated brine toward the edge of the evaporation substrate. Encouraged by this, we further designed a evaporator device to simultaneously achieve the freshwater production and directional salt collection, showing great promise for self‐sustainable agricultural irrigation (Figure 1b). Notably, the desalination device effectively circumvents salt re‐fluxing to the bulk solution, while the bottom substrate diverts re‐fluxing saline flow away from the bottom of evaporator, resulting in the directional crystallization of concentrated salts around the bottom substrate. Under prolonged 1‐sun exposure (1 kW m^−2^), sustained evaporation performance was validated across hypersaline environments (10–25 wt.% NaCl) through 145 h continuous operational trials. The mycelium‐based evaporator achieves an average evaporation rate of up to 8.1 kg m^−2^ h^−1^, a high salt collection rate of 1.95 kg m^−2^ h^−1^, a superior salt collection efficiency of 97.7%, and zero liquid discharge desalination. The evaporator device also exhibits an ultrahigh freshwater production rate of 6 kg m^−2^ h^−1^ and daily freshwater production of 32 L m^−2^ under natural sunlight. Finally, we present a self‐sustainable desalination‐irrigation system based on the evaporator device for rice growth, highlighting its potential for agricultural irrigation in remote areas. Notably, this mycelium‐based evaporator is exclusively sourced from natural ecosystems, embodies biocompatibility, renewability, and circular sustainability. Its fabrication process eliminates chemical additives and energy‐intensive processing, brings positive prospects as far as sustainability and carbon footprint.

## 2 Results and Discussion

### 2.1 Construction and Characterization of Mycelium‐Based Evaporator

The fruit body of *Bracket fungi* appears elliptical shape and has a hard, dense wood‐like shell (Figure S1, Supporting Information), whereas the other side shows a flat surface with evenly distributed pores. The longitudinal cross‐section of the fruit body further reveals the inner hierarchical structure with two distinct macroscale layers, including the middle foam‐like layer and the adjacent tubular structure layer. This double‐layered combination of mycelium facilitates the transportation of nutrients and water along the transverse and longitudinal aligned hyphae. In addition, the bottom pores are portals to tubes that extend all the way across the structure up to just beneath the middle layer. The tubular aligned channels with open pores facilitate the delivery of the spores out of the fruit body. Inspired by its functional properties, the mycelium‐based evaporation device was constructed, which composed of mycelium‐based evaporator, bottom substrate, cotton strip, and container (Figure 1). A piece of mycelium assembly can be readily prepared by cutting the hard wood‐like upper shell of *Bracket fungi*. The nylon mesh was selected for the directional transfer and crystallization of concentrated salts, and was placed on the container. The cotton strip with the end extending to the bulk water was placed between the nylon mesh and the container, ensuring adequate water supply to the evaporator. Finally, the mycelium was placed on the nylon mesh, with the tubular layer facing the sunlight. With this design, the bulk water can be transmitted through the cotton strip to the bottom foam‐like layer first and then to the tubular structure, so as the steam evaporates into the atmosphere under the sunlight illumination, mimicking the delivery process of the spores out of the fruit body.

**Figure 2:**
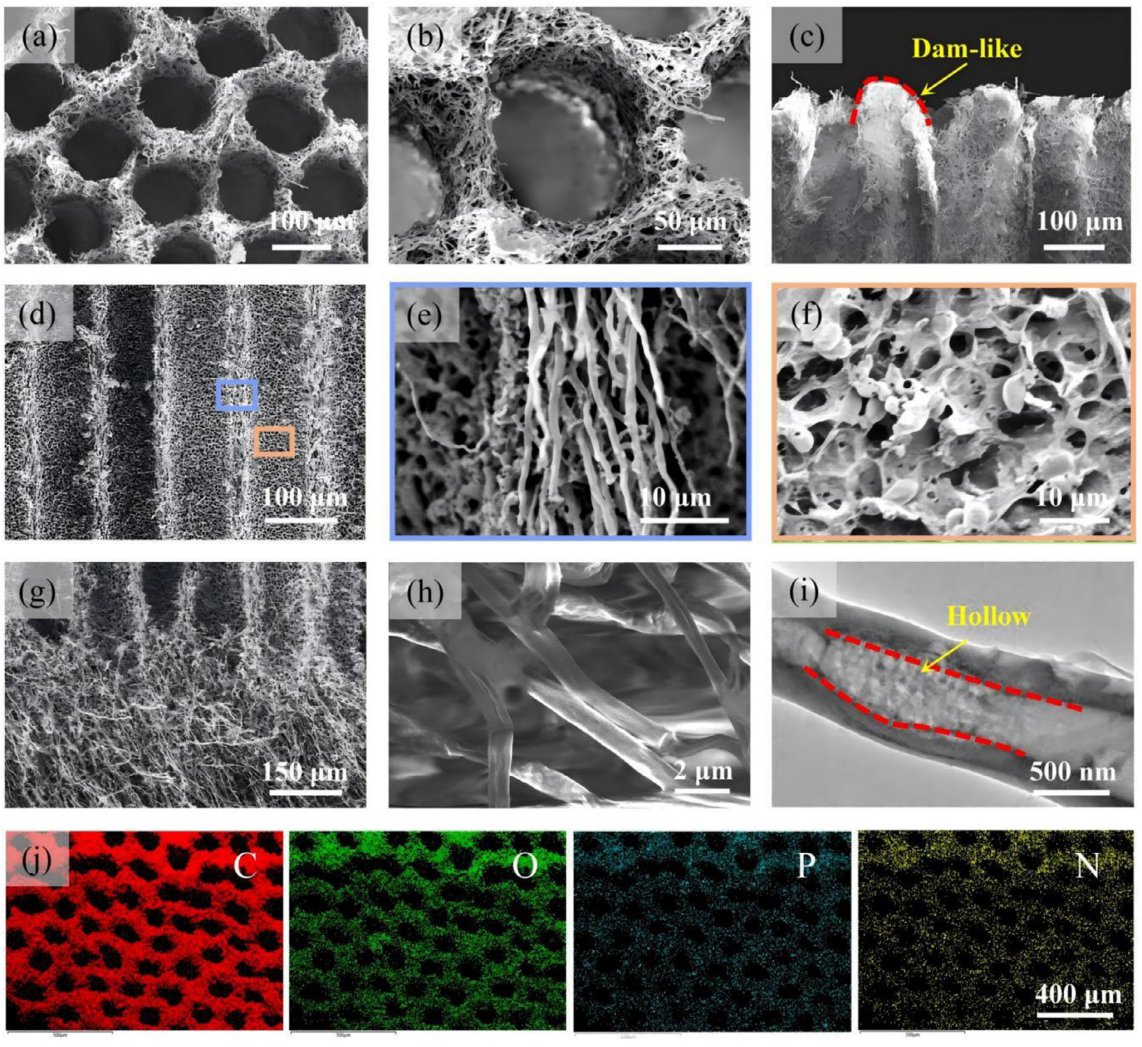
Microstructure characterization of the hierarchical mycelium. a) Top‐view SEM image of the mycelium. Partial magnified SEM image in (a) showing b) the tube pore. The longitudinal sectional SEM image of the top c) and the middle mycelium d). Partial magnified SEM images in (d), showing e) the longitudinal oriented hyphae skeleton, and f) the micron channel wall. g,h) The bottom longitudinal section SEM images, showing the transversely oriented hyphae layer. i) The HRTEM image of hyphae featuring hollow structure. j) Top‐view elemental mapping images of the hierarchical mycelium.

The microstructure of the mycelium was examined using SEM. As shown in **Figure** **2**, in contrast to the previously reported disordered and anomalous porous structure of biomass‐based hydrogel, the mycelium exhibits well‐organized double‐layered hyphae networks featuring vertically aligned tubular microchannel array (Figure 2a–d; Figure S4, Supporting Information), and the bottom foam‐like tissue (Figure 2g). The diameter of microchannel is ≈100 µm and the tube holes is surrounded by the hyphae network in a horizontal order (Figure 2b; Figure S5, Supporting Information). The longitudinal section SEM image distinctly reveals the dam‐like top structure of the hyphae skeleton (Figure 2c), which is expected to promote the solar‐light absorption and the reflux of concentrated brine. The vertically aligned tubular channels, as well as the longitudinal section of the skeleton that seamlessly connected to the bottom spongy tissue can be observed (Figure 2d–g). The partial magnified SEM image shows that skeletal hyphae formed a dense and entangled fiber bundles with a predominant orientation parallel to the long axis of the tubes (Figure 2e). Further morphology characterization shows the dense channel wall (Figure 2f), forming a thermal barrier between the micro‐channel and the hyphae skeleton, which is essential for mitigating the heat loss during the water transportation. The spongy base is connected to the vertical skeleton via an extensive network of hyphae (Figure 2g), contributing to a fast water transport. Different from the vertical hyphae skeleton, the bottom hyphae network exhibits a slightly predominant horizontal arrangement (Figure 2h), which is favorable for the transverse transportation of concentrated brine. In addition, the HRTEM image shows that the single hyphae exhibit a hollow structure. In particular, these elements, including vertical micro‐channel, hyphae skeleton, as well as the nano‐sized hollow hyphae, correspond to triple spatially low‐tortuosity channels. With the merits of the multi‐tubular structure and the natural hydrophilicity, the evaporator is expected to possess good thermal insulation and rapid water transfer. As shown in Figure 2j, energy dispersive spectroscopy results demonstrated the uniform elements distribution of C, O, P, and N within the mycelium.

**Figure 3:**
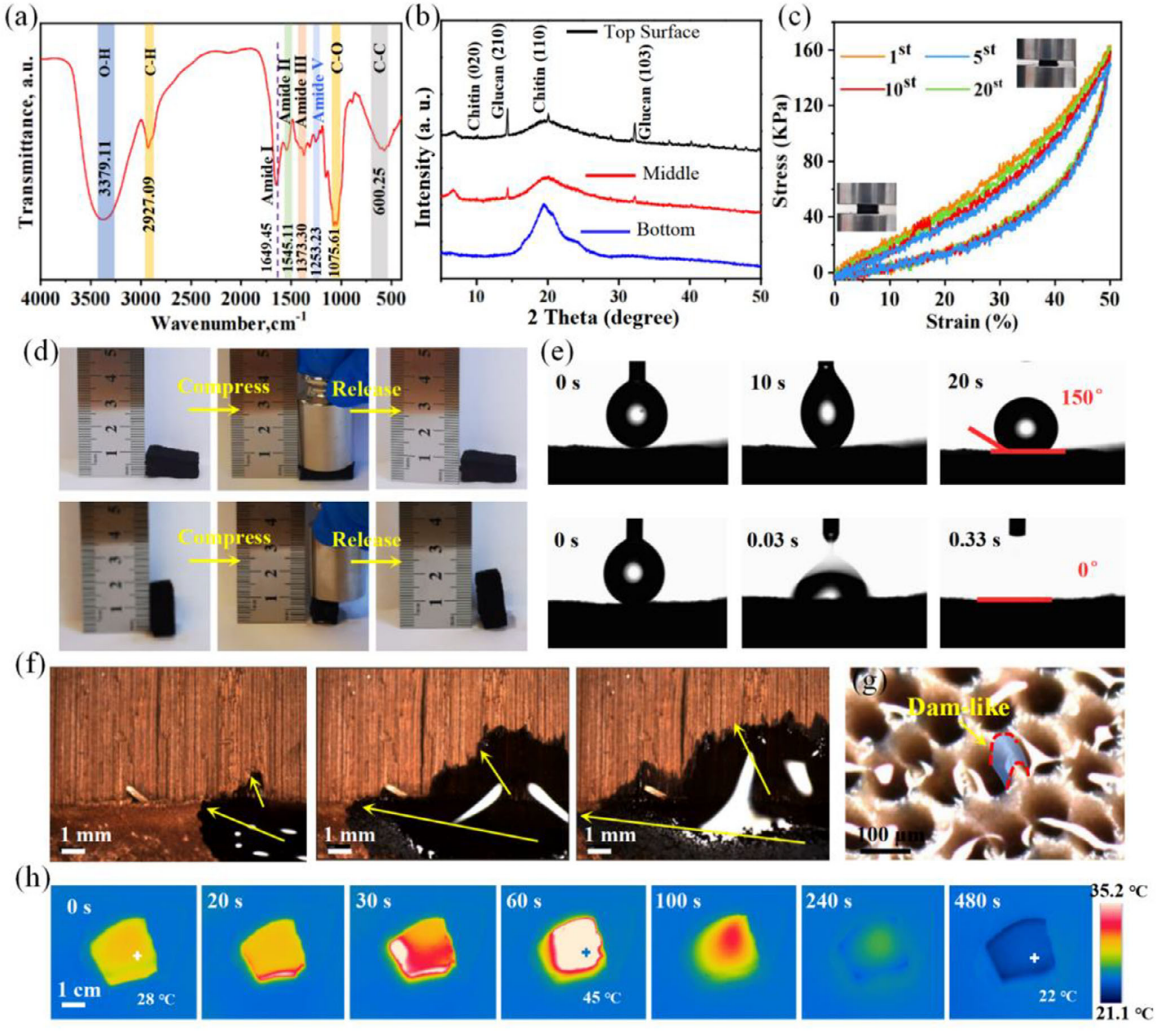
Physical, mechanical, wetting, and water transport properties of the mycelium. a) FTIR spectra, b) XRD patterns, c) stress–strain curves for 20 cycles, and d) mechanical compressibility and elasticity of the mycelium. e) Dynamic changes of the water contact angle on the surface and the bottom of the mycelium. f) The water transporting process of the double‐layered mycelium. g) The top‐view optical microscope image of the wetted mycelium. h) The infrared thermal images of the mycelium during the wetting process.

FTIR and XRD were conducted to further analyze the chemical properties of mycelium. The FTIR spectrum of mycelium is depicted in **Figure** **3**a. The characteristic peak observed at 3379.11 cm^−1^ corresponds to the stretching vibration of the O─H bond. Additionally, the peak at 2927.09 cm^−1^ is attributed to the C─H stretching vibration. A single absorption peak at 1649.45 cm^−1^ corresponds to the C─O stretching vibration of the amide I group. Weak absorption peaks at 1545.11 and 1373.30 cm^−1^ correspond to the amide II and amide III moieties, respectively. The peak amide IV group appears at 1253.23 cm^−1^. The bands at 1075.61 and 600.25 cm^−1^ correspond to C─O bond stretching and C─C bond stretching vibrations, respectively. These characteristic peaks can be attributed to chitin,^[^ ^38^ ^]^ which chemically resembles cellulose while one of the hydroxyl groups on each monomer is replaced by an acetyl amino group. The XRD patterns of the upper surface, middle part of tubular mycelium, and the bottom mycelium are illustrated in Figure 3b. The upper surface of mycelium exhibits four strong characteristic peaks at 9.71, 19.32, 14.45, and 32.41, corresponding to the (020) plane and the (110) plane of chitin, as well as the (210) plane and the (103) plane of glucan, respectively.^[^ ^39^ ^]^ The result confirms the existence of crystal structure, which could be built by the hydroxyl groups, amino groups and acetyl amino groups in the molecule that forming numerous hydrogen bonds within the cell walls. Meanwhile, the intensity of the above peaks decreased from the upper surface to the middle mycelium, and even disappeared for the bottom. The asymmetric crystallinity of the mycelium implied the gradient wettability of the multi‐leveled mycelium containing different amounts of hydrophobic and hydrophilic functional groups.

The mechanical strength and elasticity of the mycelium are critical to achieve the long‐term durability of solar steam generation for practical applications. The compressive elasticity and flexibility of the wet mycelium were further evaluated. The stress–strain compression cycles test shows that the wet mycelium maintains 93% of the initial compressive strength after 20 cycles without obvious fractures or plastic deformation (Figure 3c), showing good compressive strength and elasticity. When the wet mycelium was subjected to the large compression, the wet mycelium exhibited significant elasticity and almost fully recovered to its original height and width after releasing external pressure (Figure 3d; Video S1, Supporting Information). The excellent mechanical property of the mycelium is mainly due to its skeleton support structure and the internal interconnected network structure. In addition, the mycelium exhibits a visible darkening when hydrated, indicating a notable enhanced solar absorption (Video S2, Supporting Information). The material maintains structural integrity and reversible color change between dry and moist states across multiple hydration‐dehydration cycles, further demonstrating its stability and durability for practical applications.

The wetting properties of the double‐layered mycelium were examined by water contact angle test on the upper surface and the bottom surface of the mycelium. As shown in Figure 3e, the upper surface and the bottom surface show the water contact angle values of 150° and 0°, respectively. A drop of water was absorbed by the bottom surface in less than 0.33 s after coming in contact with the surface, while the water droplets on the upper surface can steadily stay for a long time. In addition, the evaporator's surface also exhibits hydrophobic properties for 10 wt.% NaCl, with an initial contact angle of 130.1° (Figure S6, Supporting Information). After 72 h of operation, the contact angle decreases to 93.2°, indicating a moderate reduction in surface hydrophobicity. These results confirm the asymmetric wettability of the double‐layered mycelium with the super‐hydrophobic upper surface and the super‐hydrophilic bottom layer, which is ascribed to the synergistic combination effects of the surficial porous structure and its chemical inhomogeneity as confirmed by the above FTIR and XRD results. Interestingly, the water droplets between the upper surface and the capillary were pulled into an ellipsoidal shape during the lowering of the lifting table. This adhesion behavior was attributed to the electrostatic adsorption between the water molecules and the hydrophilic functional groups within the hyphae. More importantly, this asymmetric wettability, in conjunction with the continuous water pathway by the multi‐channel structure, is expected to enable a good salt resistance and rapid water transfer.

The water transfer and absorbing of the mycelium was investigated. As shown in Figure 3f, water is preferentially transported horizontally along the bottom mycelium, and then vertically upward to the upper mycelium. This demonstrates that the water transporting direction is along the hyphae, and the continuous hyphal network can speed up water transport from the underlying mycelium to the vertically aligned mycelium, and thus avoid salt accumulation. Figure 3g shows the top‐view optical microscope image of the mycelium, distinctly revealing the wetted surface and the dam‐like tube wall. Importantly, the gentler slope at the upstream section of the dam‐like tube wall effectively regulates the water flow over the evaporative surface, thereby maintaining efficient photothermal management. In contrast, the steeper slope at the downstream section enhances structural stability while facilitating the back flow of concentrated brine, which prevents salt crystallization on the evaporation surface. Additionally, the inclined surface of the tube pore promotes sunlight absorption and enables multiple scattering of light. The saturated water content was 3.7 g g^−1^ for the samples with different area sizes (Figure S7, Supporting Information), indicating the homogeneity of the mycelium structure. The infrared thermal images of the mycelium submerged in water demonstrated the water transport pathway from the base to the upper central region of the mycelium (Figure 3h). Due to the electrostatic interaction between water molecules and hydrophilic functional groups, the surface exhibited a rapid temperature rise during water absorption. Notably, the wetted mycelium exhibited a temperature deficit relative to ambient conditions. This thermal gradient enables the low‐temperature region to harvest environmental energy, which can boost evaporation rate. Moreover, it is anticipated that concentrated brine will reflux downward through vertical micro‐channels, while the spatially isolated multi‐layered structure effectively minimizes thermal loss. Additionally, the horizontal transport characteristics of the bottom layer promote dispersion of the refluxed brine toward the edge of the bottom substrate.

### 2.2 Solar Absorption of Mycelium‐Based Evaporator

**Figure 4:**
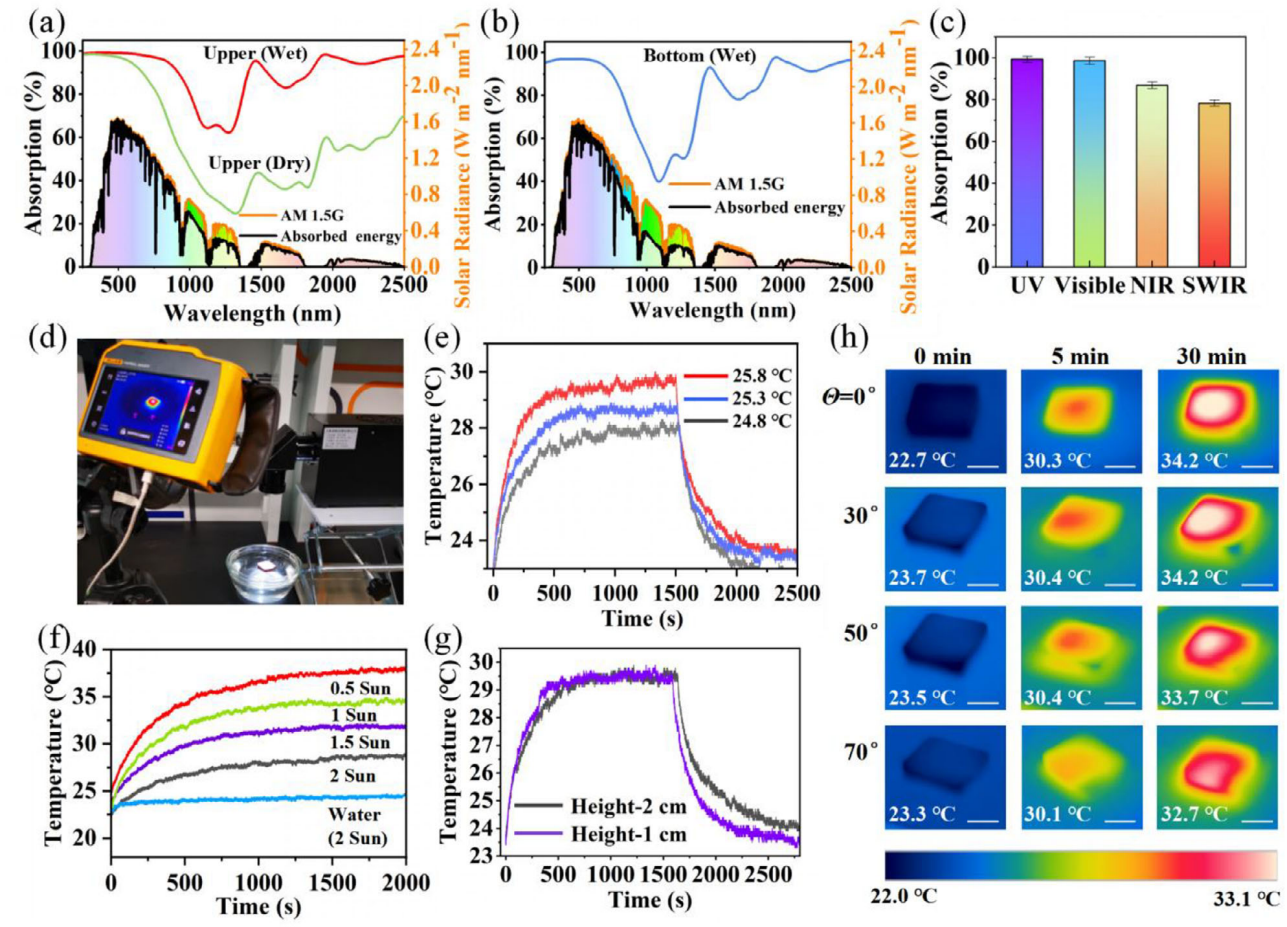
Photothermal conversion and thermal localization of solar evaporator. UV–vis–NIR spectra of a) the upper surface and b) the bottom surface of the mycelium‐based evaporators. The background in (a) and (b) is the normalized spectral solar irradiation of the air mass 1.5 global (AM 1.5G) and the absorbed solar energy of the corresponding samples. c) The average absorption property of the upper surface of wet evaporator in the ultraviolet (UV), visible, near‐infrared (NIR), and short wavelength infra‐red (SWIR) spectrum. d) Photographs of the solar evaporation tests. The surface temperature changes corresponding to e) different ambient temperature, f) various sunlight irradiation intensities, and g) different heights of samples under 1 sun illumination. h) Infrared thermal images of the evaporator at different angles of incidence under 1.5 kW m^−2^ irradiation. (Scale bar: 2 cm).

The solar absorption properties of the evaporator were assessed based on UV–vis and UV–vis–NIR measurements. The integrated absorptance based on first‐law balance was used to evaluate the solar energy utilization efficiency of the hierarchical evaporator (Note S1, Supporting Information). As illustrated in **Figure** **4**a, the upper wet surface exhibits a high light absorption of 92.1% from 250 to 2500 nm. In contrast, the upper surface of dry sample exhibits 74.7% absorbance of the standard solar spectrum (AM 1.5G) (Figure S8, Supporting Information). The lower light absorption of dry sample was attributed to the increase in light reflectivity for the air‐solid interface. The outstanding light absorption of the upper wet surface results from the combination of nanocrystalline structure and the multiple reflection within the vertical channels, which can be evidenced by the relatively lower average light absorption of 83.2% as for the bottom layer without micron channels (Figure 4b). The high solar light absorption property of upper surface of mycelium endowed the evaporator with outstanding interfacial photothermal conversion properties. Correspondingly, the upper surface of wet evaporator had average absorption 99.22%, 98.61%, 86.82%, and 78.28% in the ultraviolet (UV), visible, near‐infrared (NIR), and short wavelength infra‐red (SWIR) spectrum, respectively (Figure 4c). The thermal conductivity of the mycelium was further evaluated in the dry and wet states using a hot wire thermal analyzer. In the wet state, the thermal conductivities of the top mycelium and the bottom mycelium were 0.2 W m^−1^ K^−1^ and 0.12 W m^−1^ K^−1^, respectively, which is lower than that of pure water (0.61 W m^−1^ K^−1^). In addition, the mycelium in the dry state exhibits an extremely low thermal conductivity (≈0.04 W m^−1^ K^−1^) owing to the unique multi‐layered channel structure. Importantly, the multi‐level channels with low thermal conductivity could act as a “barrier” to heat transfer, thereby endow the evaporator with excellent thermal insulation and good photothermal localization.

To evaluate the photothermal conversion abilities, the surface temperatures of mycelium were monitored using IR thermal imaging, as shown in Figure 4d. During the solar illumination, the ambient temperature had an influence on the surface temperature of evaporator (Figure 4e). In order to investigate the surface temperature change under varying solar intensities, the following experiment was carried out at constant temperature. Under one sun (1 kW m^−2^) illumination, the surface temperature of wet mycelium‐based evaporator increased quickly to over 30 °C at 10 min and eventually reached a steady temperature at 32 °C within 30 min, indicating its good photothermal conversion performance (Figure 4f). The surface of evaporator demonstrated consistent temperature increases as solar radiation intensity augments, reaching 28.8, 32, 34.2, and 38 °C, respectively. In contrast, the temperature of the bulk water remains ≈24.5 °C even under two sun (2 kW m^−2^) illumination. In addition, the surface temperature remains constant as the evaporator height increases (Figure 4g). These results demonstrated the excellent thermal localization performance of the mycelium. Previous studies have demonstrated that the unicity of water channels induce significant heat loss. In this work, the spatially multi‐layered vertical channels, as well as the bottom spongy‐like fibers contribute to the excellent photothermal management. To investigate the directional dependence of the solar absorbers, the surface temperature of the evaporator at different angles of incidence were measured. The infrared images reveal that the surface temperature of the evaporator under 1.5 kW m^−2^ irradiation for 30 min shows a slight decrease from 34.2 to 32.7 °C when the incident angle increases from 0° to 70° (Figure 4h). The trapezoidal dam‐like tube walls aid in multi‐angle light absorption, thus contributing to the low dependency of solar‐thermal conversion on light direction. The excellent photothermal conversion and thermal localization performance, as well as the low dependency on light direction highlights the mycelium's potential in highly efficient solar‐driven evaporation.

### 2.3 Directional Salt Collection and Zero Liquid Discharge Desalination of High‐Salinity Brine

**Figure 5:**
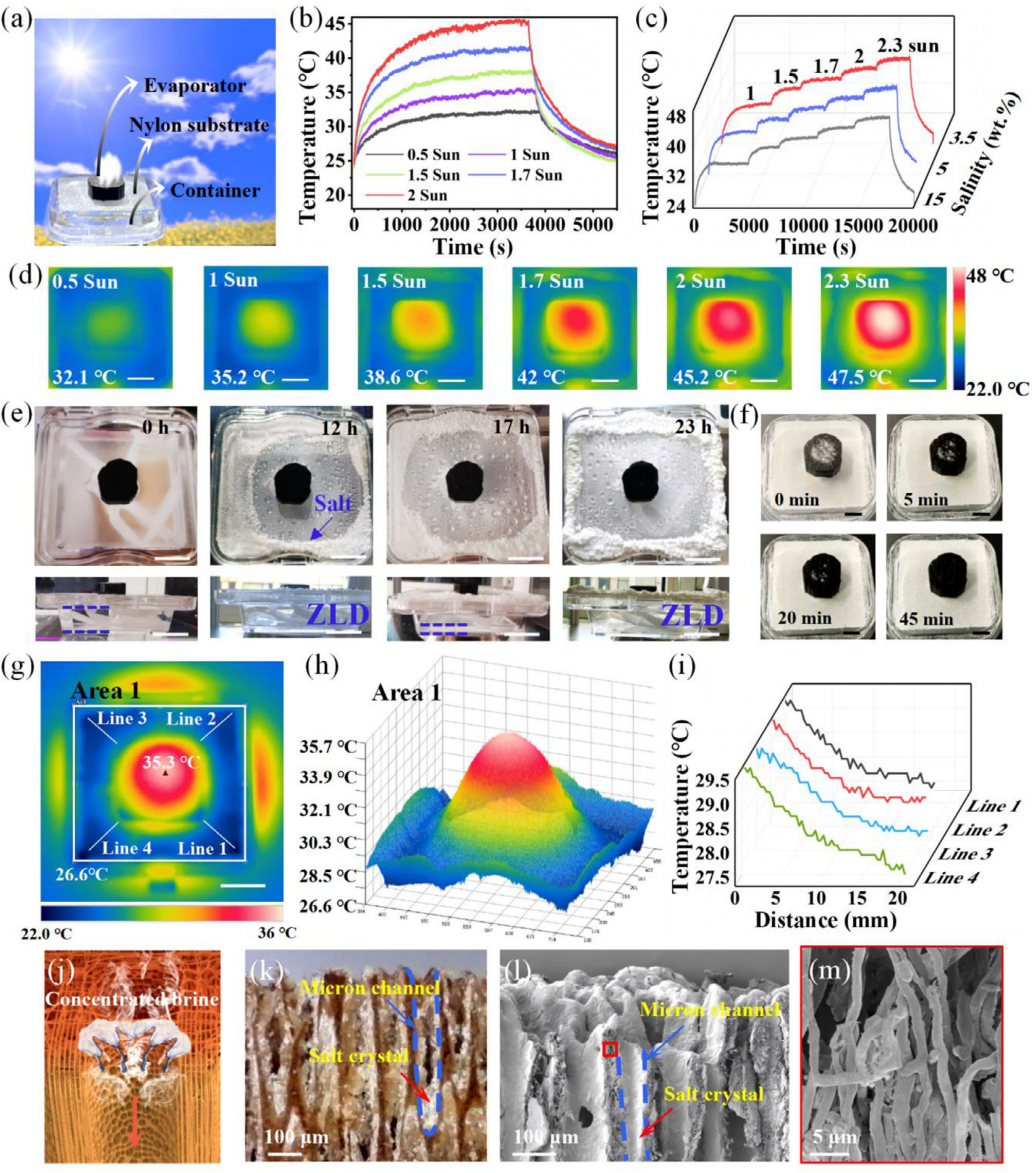
Construction of the mycelium‐based desalination device enabled the directional salt collection and zero liquid discharge desalination. a) Scheme of the evaporator device setup for the directional salt collection. b) The surface temperature at 10 wt.% brine under varying solar intensities. c) The consistent temperature trends at different salinities under cycling gradually enhanced solar intensities. d) Infrared thermal images of evaporation device under varying solar intensities. (Scale bar: 2 cm) e) Photographs of the evaporation device during a continuous 23 h desalination test under natural sunlight. (Scale bar: 2 cm) f) Time‐independent salt dissolution process on the surface of the evaporator. (Scale bar: 1 cm) g) The top‐view infrared thermal image of evaporation device during desalination. (Scale bar: 2 cm) h) The 3D view of the selected area in Figure 5g. i) The temperature distributions along the lines in Figure 5g. j) Schematic showing the re‐fluxing concentrated brine within micron tubular channels. The longitudinal sectional optical microscope image k), and SEM image l) of the mycelium employed for desalination, distinctly reveal the deposited salt crystals within the micron channels. (m) Partial magnified SEM image in (l), showing the porous hyphae skeleton.

Salt accumulation on the interface is a major challenge for achieving zero liquid discharge desalination of high‐salinity brine. Benefiting from the vertical micron channels, the concentrated brine at the evaporation interface can re‐flux downward, while the bottom layer promotes the brine transfer toward the edge of the evaporator. (Figure S9, Supporting Information). Inspired by this phenomenon, we designed a mycelium‐based evaporation device (MDs) to achieve the directional salt crystallization and collection, as shown in **Figure** **5**a. The nylon substrate was placed underneath the evaporator, which was served as the salt collection substrate for the transfer and crystallization of concentrated salts. The re‐fluxed brine will flow from the bottom of the evaporator to the edge of substrate. During this process, the vapor at the gas‐solid interface will take away the heat, and the concentration of the brine continues to increase until it reaches saturation, leading to directional salt crystallization on the substrate.

The directional salt collection tests were performed using an artificial brine solution (100 mL) with 10 wt.% NaCl. The surface temperature change was monitored by IR camera under different intensities of solar irradiation, and the effect of varying brine concentrations on the surface temperature of evaporator was explored. At 10 wt.% brine, the stabilized surface temperatures were 31.5, 35.2, 38.6, 42 and 45 °C with uniform surface temperature variations when the solar radiation intensity was 0.5, 1, 1.5, 1.7 and 2 sun (Figure 5b). Meanwhile, the consistency of temperature trends at different salinities under cycling gradually enhanced solar intensities further indicate that the evaporation device exhibits sensitive and steady evaporation processes (Figure 5c,d). As shown in Figure 5e, the evaporation device achieves complete separation of water and salt from 10 wt.% brine in a continuous 12 h desalination process, resulting in ZLD. Salt crystallization was clearly observed around the nylon substrate, and no salt accumulation occurred on the evaporator surface (Figure S10, Supporting Information). We further sprinkled salt crystals on the upper surface of the evaporator to observe salt dissolution behaviors (Figure 5f; Video S3, Supporting Information). The result shows that salt crystals were completely dissolved within 45 min, confirming the excellent salt‐resistance performance. After the first cycle of ZLD solar evaporation, the container was refilled with the same amount of brine solution and restarted the solar evaporation. The second cycle was completed within 11 h, suggesting the steady evaporation process. Interestingly, the salt crystals initially formed on the nylon substrate and gradually migrated outward, and finally deposited on the peripheral of the nylon substrate (Figure S11, Supporting Information). The enlarged image shows that the salt crystals were loose and porous, it is assumed that the brine could diffuse across and push forward the as‐formed salt crystals for further deposition and accumulation. As a result, the directional transfer and deposition of the salt crystals ensure the stable salt collection.

To depict the directional crystallization mechanism of concentrated salts, the temperature distribution of the MDs during desalination was investigated by IR camera. As shown in Figure 5g, the surface temperature of the salt layer on the nylon substrate is lower than the ambient temperature due to the evaporative cooling effect of the substrate. The 3D view of the selected area in Figure 5g shows a continuous gradient temperature distribution along the radial direction of the substrate (Figure 5h), which aligned with the transfer path and directional crystallization of the brine from the evaporator to the outer edge of the substrate. As the brine spread outward, the salt solubility gradually decreased under the cooling effect of the substrate until the salt crystals had precipitated. The advancing saline then pushed forward these crystals to spread further while continuing migration until crystallization occurred. Since the crystallization process released heat, the continuous diffusion and crystallization process created an evenly distributed temperature gradient on the substrate (Figure 5i). To reveal the reflux of the concentrated brine during the desalination (Figure 5j), the longitudinal sectional optical microscope and SEM images of the mycelium employed for desalination were investigated (Figure 5k,l; Figure S12, Supporting Information). As indicated by the red arrow, the vertical micron channels and the bottom interstices of the hyphae network were deposited with salt crystals, while the hyphae skeleton remained porous structure without salt blockage (Figure 5m). These results corroborate the collaborative effect of functional sub‐regions based on a multi‐level tubular structure. Brine was transported upward through the hyphae skeleton, while the concentrated brine flowed downward through vertical micron channels, with the underlying structure facilitating the radial dispersion of the concentrated brine. This synergistic effect not only enables directional salt deposition, but also endows the evaporator with excellent salt‐resistance and long‐term stability.

### 2.4 High‐Efficiency Vapor Generation and Long‐Term Stability

**Figure 6:**
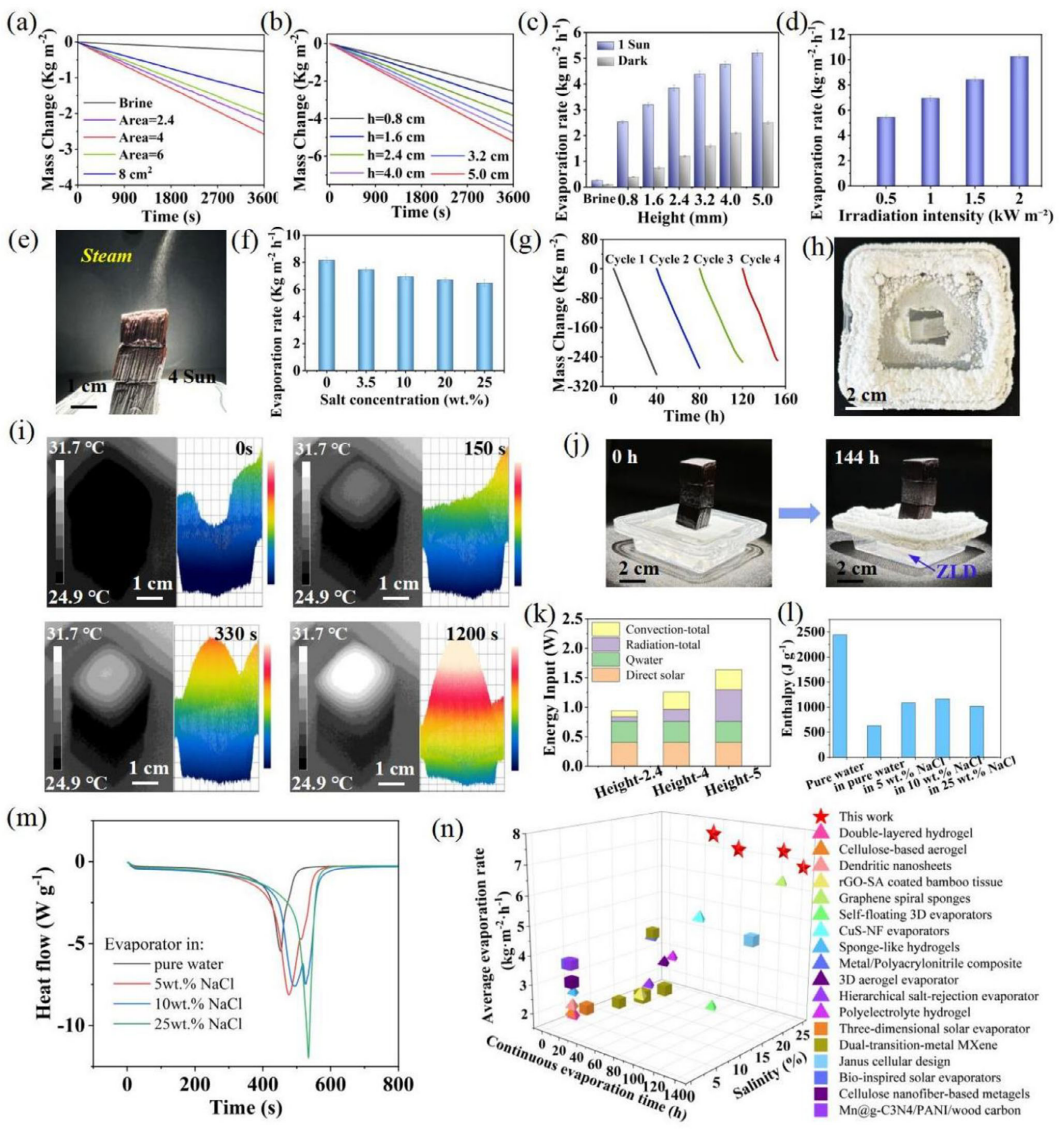
High‐efficiency vapor generation and long‐term stability of the MDs. Mass change curves of 10 wt.% brine under 1 sun solar irradiation for a) brine and evaporators with top area varying from 2.4 to 8 cm^2^, height is 0.8 cm, and b) evaporators with height varying from 0.8 to 5.0 cm, area is 4 cm^2^. c) Evaporation rates of 10 wt.% brine and evaporators with different heights under 1 sun illumination and dark conditions. d) Evaporation rates of 10 wt.% brine under varying solar intensities for evaporator with top area=4 cm^2^, height=5 cm. e) The photograph of the evaporation device during desalination test under 4 sun illumination. f) Evaporation rates of the MDs for brines with different salt concentrations under 1 sun illumination. g) Mass change curves of brines with varying salt concentrations over the 145 h ZLD solar evaporation tests. h) The top‐view of the MDs after the ZLD desalination test. i) The temperature evolution on the top and side surface of evaporator under 1 sun illumination. j) Photographs showing the salt directional crystallization over a continuous 145 h desalination test. k) Contribution to the energy input from direct solar, radiation, convection and diffusion in the evaporators. l) Evaporation enthalpy of the pure water and the evaporators in pure water, and 5, 10, 25 wt.% NaCl. m) DSC curves of the evaporator in pure water, 5, 10, and 25 wt.% NaCl. n) Comparisons of solar desalination performance.

The heat transfer process for solar‐driven steam encompasses solar energy capture, steam generation, and environmental dissipation. It has confirmed that the 3D structures can effectively improve the solar‐to‐vapor conversion efficiency by increasing the evaporation area and collect environmental energy.^[^ ^22^, ^40^ ^]^ In this paper, the vertical channels with dam‐like and porous tube wall enable multiple light reflection, meanwhile, the side surface of the mycelium can exchange heat with the surrounding environment, which is expected to enhance the evaporation efficiency of the evaporator (Figure S2, Supporting Information). To investigate the evaporation performance, the evaporators with varying area and height were prepared, and their evaporation rates were evaluated in 10 wt.% brine and compared to 10 wt.% brine without an evaporator. We precisely defined the illumination zone to ensure that incident light only covers projected area (Figure S15, Supporting Information), thereby preventing direct exposure to container walls or surrounding water bodies (Video S4, Supporting Information). The experiments were conducted in a controlled environment with constant temperature (28 °C) and constant humidity (50%) to minimize the effects of environmental changes. As depicted in **Figure** **6**a, the cumulative mass loss of 10 wt.% brine was only 0.26 kg m^−2^ after 1 h under one sun irradiation. In contrast, water evaporation of evaporators with areas of 2.4, 4, 6 and 8 cm^2^ achieved cumulative mass losses of 2.23, 2.58, 2.04, and 1.44 kg m^−2^, respectively. Among them, the evaporator with an area of 4 cm^2^ exhibited the highest evaporation rate. In addition, the evaporation rate of the evaporator is positively with the side height, and is much higher than the 10 wt.% brine both under 1 sun illumination and dark (Figure 6b,c). In particular, the evaporator with a side height of 5 cm achieved the highest evaporation rate of 5.55 kg m^−2^ h^−1^. This improvement is due to the increased evaporation area of the side surface, which effectively enhanced the environment energy acquisition and reduced heat loss (Note S2, Table S1, Supporting Information), and the evaporation efficiency is significantly exceeded the theoretical limit, reaching values of 241% for the evaporator with height of 5 cm (Note S3, Table S1, Supporting Information). The result also demonstrates that the evaporator can easily transport water up to a height of 5 cm. The favorable water transfer behavior could be mainly attributed to vertically aligned capillary channel, contributing to a faster water transport compared to the disordered porous structure.

The evaporation rate of the MDs is positively with the intensity of solar radiation (Figure 6d). Even under 0.5 sun illumination, the MDs still maintained a high evaporation rate when dealing with 10 wt.% brine. This is essential for practical applications in the case of insufficient solar irradiation. Under the illumination of 4 sun, steam is clearly observed escaping continuously from the evaporator surface (Figure 6e; Figure S13, Supporting Information). This phenomenon further confirms the excellent water transfer of the MDs. In addition, the MDs can be used to treat brine concentrations ranging from 0 to 25 wt.% (Figure 6f). Even for 25 wt.% brine, the evaporation rate was still as high as 6.47 kg m^−2^ h^−1^ under 1 sun illumination, indicating the stable and reliable solar‐driven desalination. The evaporation rate exhibits an enhancement on airflow velocity, with higher rates observed under 33 °C compared to 28 °C, both under 1 sun illumination and dark conditions (Figures S16 and S17, Supporting Information). The evaporator achieved a maximum rate of 7.57 kg m^−2^ h^−1^ at 33 °C and 0.4 m s^−1^ airflow, demonstrating synergistic enhancement from convective mass transfer. Under relatively lower humidity and higher temperature conditions, the evaporation rate further increases (Figure S18, Supporting Information), which drives more rapid vapor diffusion.

To evaluate the long‐term stability and durability of the mycelium‐based evaporator, the evaporator device was continuously used to treat 10 wt.% NaCl solution for three cycles and subsequently 25 wt.% NaCl solution for one cycle under 1 sun illumination. As shown in Figure 6g, the mass change of the evaporator device for each cycle of ZLD solar evaporation remains linear with time, and the average mass change is 270 kg m^−2^. In addition, the evaporation rate for the initial two ZLD cycles remains relatively stable overall (Figure S19, Supporting Information), with minor fluctuations attributed to variations in ambient temperature and humidity. Notably, the evaporation rate increases sharply for the initial hours of the subsequent two cycle. For example, the evaporation rate increased from 7.05 to 10.25 kg m^−2^ h^−1^ for the fourth cycle. The average evaporation rate is 7.19 and 8.1 kg m^−2^ h^−1^ for the 10 and 25 wt.% NaCl solution, respectively. The increased evaporation rate for the 25 wt.% NaCl is primarily attributed to the formation of abundant intermediate water within the hydrated network of the biomolecular (Figure 6m; Figures S21–S23, Supporting Information). After ZLD desalination, the salt crystals deposited on the substrate can be clearly observed (Figure 6h–j). The periodic enhancement in solar evaporation rate is dominated by augmented interfacial evaporation areas stemming from rewetted dendritic salt crystals (Figure S14, Supporting Information). The fourth cycle of ZLD solar evaporation was completed within 32 h. The steady evaporation process confirm that the evaporator device can implement long‐term and stable ZLD solar evaporation of high‐salinity brine.

Salt accumulation on the surface of evaporator is a common challenge for most solar desalination devices, which reduces evaporation efficiency and impedes steam generation stability. The subsequent physical salt‐scraping compromises structural integrity, impairing continuous long‐term functionality. In contrast, the as‐designed desalination device can overcome this challenge and maintains consistent high evaporation efficiency without material integrity compromise even at high concentration brine (Figure 6h). The excellent salt resistance can be attributed to the fast capillary transport benefiting from the vertical capillary skeleton (Figure S2, Supporting Information), and the Marangoni convection by the temperature gradient (Figure 6i; Figure S3, Figures S24 and S25, Video S4, Supporting Information). Moreover, the residual salt crystals within the evaporator at the end of the ZLD solar evaporation can be easily dissolved (Figure S26, Supporting Information), showcasing its promising potential for practical seawater desalination. From the top‐view of the desalination device, all the salt crystals accumulated around the nylon substrate, while the bottom of the evaporator was free of salt crystals (Figure 6h), confirming the bottom horizontal transport of the refluxed concentrated brine, which contributing to the ZLD solar evaporation.

For practical applications, most evaporators suffer from frequent salt‐shedding cycles requiring labor‐intensive dismantling/cleaning, which is not economical and inefficient, especially for scaled‐up solar seawater desalination. In this study, the deposited salt crystals could be easily removed and collected (Figure S27), achieving a high salt collection rate of 1.95 kg m^−2^ h^−1^ and salt collection efficiency of 97.7%. The desalination device showed superior evaporation efficiency and salt collection performance, outperforming other reported solar desalination systems (Figure 6n; Table S2, Supporting Information).^[^ ^10^, ^13^, ^17^, ^41^, ^42^, ^43^, ^44^, ^45^, ^46^, ^47^, ^48^, ^49^ ^]^ Furthermore, the evaporator exhibits long‐term cyclic fatigue resistance, biofouling resistance, and UV stability (Figures S28–S30, Video S5, Supporting Information). To explore the practical applicability of the evaporator, Rhodamine B (RhB, 1 mg L^−1^) was chosen as representative dye pollutant. The top of the container was covered with a transparent glass cover, while the bottom featured a culture dish to collect the fresh water. During the evaporation process, the water vapor condensed into droplets on the inner surface of the glass cover, subsequently flowing into the culture dish below (Figure S31, Supporting Information). After purification by the evaporator, the pink (RhB) solution turned transparent. The UV–vis spectra confirmed that the dye removal rate in the purified water reached nearly 100%, showcasing its potential for practical wastewater treatment applications.

### 2.5 Outdoor Seawater Desalination and the Cultivation System

**Figure 7:**
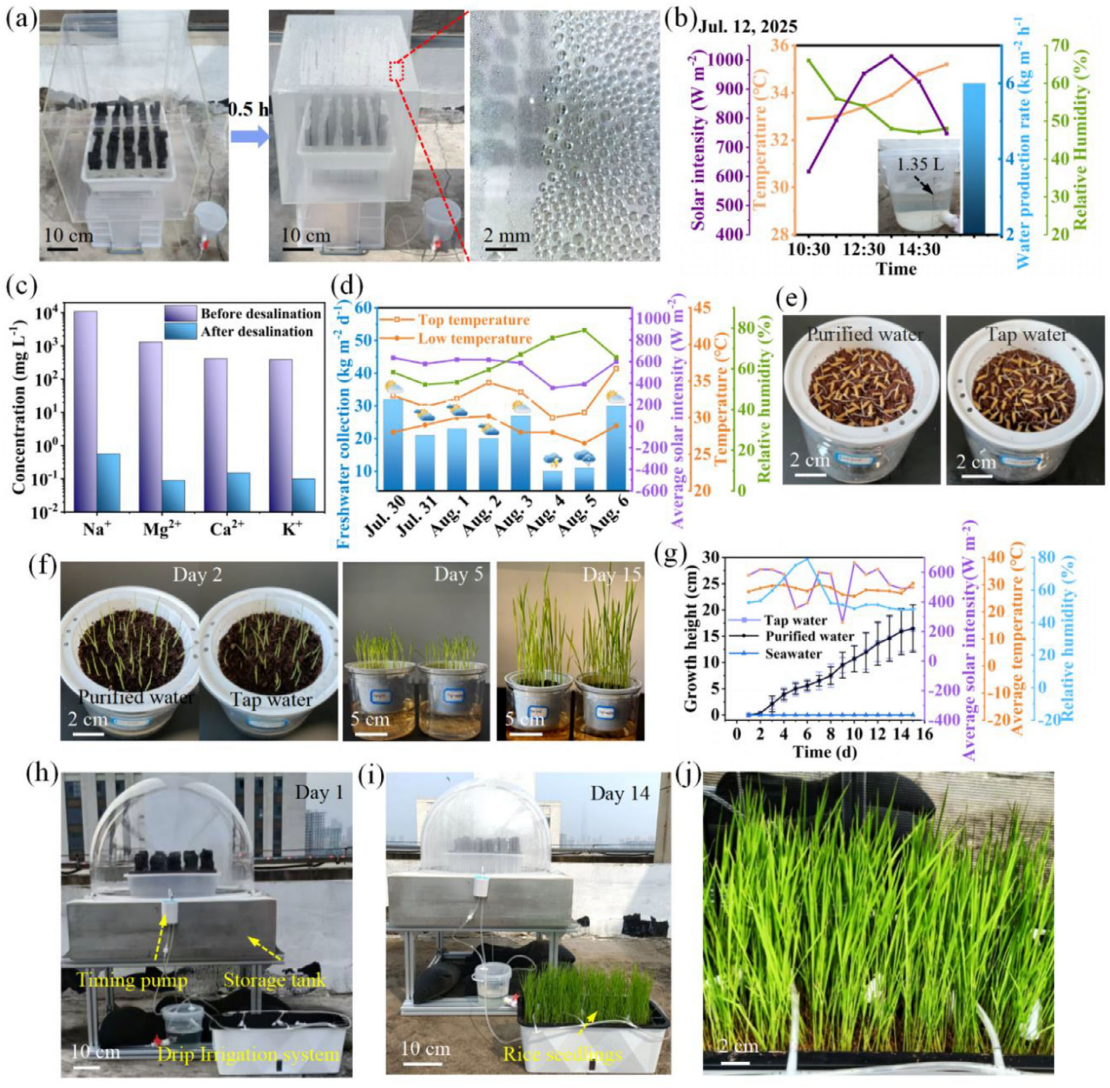
Continuous freshwater production of evaporators and rice breeding applications. a) Digital photos of the desalination system. After sunlight irradiation for 0.5 h, the resulting freshwater droplets flowed continuously. b) The water collection rate and conditions during the outdoor testing. The inset image shows the photograph of the freshwater collected from 10:00 AM to 15:00 PM. c) The measured concentrations of primary ions in a real seawater sample (from Bohai, China) before and after solar desalination. d) The water collection rates and conditions during the outdoor testing. e) The germination and f) the growth progression images of rice irrigated with purified water and tap water, respectively. g) Time‐independent growth height of rice seedlings irrigated with different water sources. h,i) Photograph of the continuous desalination‐irrigation platform. After 14 days of irrigation, the rice seedlings exhibited vigorous growth. j) The enlarged image of the growing rice seedlings.

The solar‐driven desalination device for freshwater production is a promising sustainable technology to alleviate world water scarcity. To evaluate the feasibility of this desalination device, real seawater (collected from the Bohai Sea, China) evaporation was tested outdoor. A desalination system based on the evaporator was designed for freshwater production (**Figure** **7**a). The ambient temperature, relative humidity, solar intensity, and freshwater production rates were recorded. As water vapor condensed, the device's surface fogged rapidly, and the resulting freshwater droplets flowed continuously into the collection container. During the 5 h of natural sunlight radiation with an average light of ≈841 W m^−2^ (from 10:30 AM to 15:30 PM), the evaporator yielded 1.35 L of freshwater, achieving an ultrahigh production rate of 6 kg m^−2^ h^−1^ (Figure 7b; Figure S32, Supporting Information). The ion concentrations (Na+, K+, Ca^2+^, and Mg^2+^) were determined to assess the quality of the collected water. The results indicated that all ionic indicators are well below the guideline values of the Ministry of Ecology and Environment of China and the World Health Organization (WHO) for drinking water (Figure 7c). The water production of the desalination device was further evaluated via 21‐day outdoor testing under varying weather conditions (Figure 7d; Figures S33 and S34, Supporting Information). The daily water production ranged between 0.36 and 1.44 L, corresponding to 8‐32 L m^−2^ per day of freshwater, which was ahead of other solar evaporators reported so far (Table S3, Supporting Information).^[^ ^50^, ^51^, ^52^, ^53^, ^54^, ^55^, ^56^ ^]^

The purified water collected from the desalination device was used for rice breeding. Rice seeds irrigated with seawater failed to germinate due to the high salinity (Figure S35, Supporting Information). In contrast, rice seeds irrigated with purified water and tap water germinated within 2 days, with similar germination rates (Figure 7e). The rice irrigated with purified water exhibited a comparable growth rate to the rice irrigated with tap water, reaching ≈165 mm in 15 days (Figure 7f,g). To reveal the potential of the developed system for large scale automatic irrigation, a scaled‐up desalination‐cultivation platform was constructed for continuous freshwater production and rice breeding (Figure 7h; Figure S36, Supporting Information). A precisely measured 300 mL of harvested water was automatically delivered to rice seedlings in the growth chamber via drip emitter arrays at 6‐h intervals, providing a cumulative daily irrigation volume of 1.2 L to the cultivation assembly. After 14 days of irrigation, rice seedlings exhibited vigorous growth with stem height reaching 18.9 ± 3.4 cm (Figure S37, Supporting Information). The integrated desalination‐cultivation platform designed in this study provides a promising solution for addressing freshwater shortages and rice cultivation challenges in saline‐alkali regions. In addition, the preparation cost of the evaporation device is as low as 1.325 USD m^−3^ (Table S4, Supporting Information), showing significant economic advantages for large‐scale application in areas where resources are limited.

## 3 Conclusion

This study presents a new solar evaporator with 3D ordered hierarchical structure for high evaporation rate and ZLD desalination of high salinity water. The evaporator leverages the synergistic structure‐chemistry coupling properties of the natural mycelium, including multi‐level spatially low‐tortuosity channels, dam‐like tube wall, and gradient wettability, addresses the long‐standing trade‐off between the water supply, salt accumulation, and thermal localization. As a result, efficient light to thermal conversion, rapid water transportation, suppression of thermal loss, and excellent salt resistance are observed. Furthermore, a desalination device is designed for directional salt collection and zero liquid discharge desalination, resolves the fundamental conflict between material degradation and aquatic ecosystem pollution. The desalination device demonstrated high and stable performance over continuous 145‐h solar evaporation tests in hypersaline environments (10‐25 wt.% NaCl), achieving average evaporation rate of 8.1 kg·m^−2^ h^−1^, high salt collection rate of 1.95 kg m^−2^ h^−1^, superior salt collection efficiency of 97.7%, and ZLD desalination. The desalination device also exhibits an ultrahigh freshwater production rate of 6 kg m^−2^ h^−1^ and daily freshwater production of up to 32 L m^−2^ during the outdoor desalination test. In addition, a continuous desalination‐cultivation platform is constructed, demonstrates the feasibility of the evaporator for self‐sustainable agricultural irrigation. This novel bio‐templated evaporator design provides promising prospects for advancing green, sustainable and high‐efficient solar evaporators with low carbon footprint for large‐scale desalination of high‐salinity brine.

## 4 Experimental Section

### Preparation Process of the Mycelium‐Based Evaporator Device

The mycelium‐based evaporator could be easily prepared from *Bracket fungi*. First, the fruit bodies were prepared by cutting down the hard wood‐like shell of the *Bracket fungi*. Then, the obtained fruit bodies were cut into pieces of mycelium with varying heights and side lengths. Finally, the mycelium was placed on a self‐made evaporation device. The evaporation device consists of a sealed container with a slit at the top, cotton strip and a piece of nylon mesh. The cotton strip was immersed in the water within the container with the other end extending out of the container through the slit. Then, a piece of nylon mesh was placed on the surface of the container.

### Material Characterizations

The microstructure and element analyses of the mycelium were characterized by a field‐emission SEM (7610F, Hitachi, Japan). TEM image of the hyphae was obtained via a JEM‐2100F microscope. Fourier infrared transform spectroscopy (FT‐IR) spectra were recorded on a total reflection Fourier infrared transform spectrometer (Nicolet 6700, Thermo Fisher Scientific Co.). The X‐ray diffraction (XRD) patterns of mycelium were collected by D8 Advance X‐ray diffractometer (BRUKER D8 ADVANCE) at a range of 2*θ*= 10–90 °. The mechanical properties of mycelium were tested at room temperature at a speed of 5 mm min^−1^ using an Electro‐mechanical Universal Testing Machine (Hengyu, UTM4203X, China), and the stress–strain curves were obtained. The water contact angle of the mycelium was measured on an OCA40 Micro Contact angle measurement system (Data‐physics). The thermal conductivity of the aerogels was characterized by a hot disk (TC3000e) based on the transient hot‐wire method. Reflectance and transmittance spectra (250–2500 nm) of the aerogels were collected on an UV–vis–NIR (UV‐3600 Plus, Shimadzu) spectrophotometer coupled with an integrating sphere. The Rhodamine B (RhB) in water was determined using a UV spectrometer (UV‐2600i, Japan). The concentrations of salt ions were measured using ICP‐MS instrument (ICP‐OES, Optima 5300 V, Perkin Elmer).

### Solar Evaporation Experiment

Solar simulator (CEL‐S500‐T5) with an optical filter for the standard AM 1.5 G spectrum was used to simulate sunlight. The size of the light spot was controlled by an aperture, ensuring that it was equal to the size of the sample. The radiation intensity of the top projected plane of the sample was corrected by an optical power meter (TES, TES‐1333R, China). The surface temperatures were recorded using an infrared thermal imager (Fluke, TiX1060, USA). Under varying solar irradiation from 0.5, 1, 1.5 to 2 kW m^−2^, the mass loss of the water was monitored in real‐time by an electronic balance, respectively. The evaporation rate was calculated by the following equation:

(1) $$v=\frac{dm}{s\times dt}$$

where *m* is the mass loss of the water, *s* is the illuminated area, *t* is evaporation time, *v* is evaporation rate.

### Long‐Term Stability Test

The long‐term zero liquid discharge evaporation was investigated under solar irradiation with 1 kW·m^−2^ in 10 wt.% NaCl solution for three cycles and subsequently 25 wt.% NaCl solution for the fourth cycle. The mass loss of water, ambient temperature and humidity were recorded throughout the experiment, and salt crystallization on the surface of the container was monitored. After the test, the mass of the collected salt was measured by an electronic balance.

### Wastewater Purification of Mycelium‐Based Evaporator

Rhodamine B (1 mg L^−1^) was used as pollutants. Purified water was collected using the evaporation‐collection device, and then analyzed via UV–vis absorption spectroscopy (TU‐1901, Purkinje, China).

### Outdoor Seawater Evaporation‐Cultivation System

A seawater desalination‐cultivation system was built by connecting an enlarged desalination device with a culture container. The desalination device was designed by placing an integrated evaporation unit with several evaporators (top projected plane: 0.045 m^2^) inside a transparent hemispherical condensing container. The top projected plane of the evaporators was used for calculating the water production rates. Seawater was taken from the Bohai Sea of China (Tianjin City). The purified water flows through the surface of the condensing container into the storage tank. The experiment was conducted outdoors at Tiankai Higher Education Innovation Park, Tianjin, China. (From July 30 to August 6, 2025). Ambient temperature, humidity, and solar intensity were measured using a thermometer, a hygrometer, and an irradiance meter.

### Rice Cultivation Process

Rice seeds were soaked in purified water for 12 h. The purified water was poured into the dry natural soil to form moist soil, which was then packed in culture containers with holes at the bottom. Rice seeds were spread evenly on the soil, and covered with 5 mm‐thick soil. During the rice seedling period, A precisely measured 50 mL corresponding water was irrigated each day to maintain soil moisture. The tap water and seawater were selected for the control experiments. The temperature, humidity, and growth height of the rice seedlings were measured daily. For the scaled‐up cultivation of rice seedlings, 300 mL of collected water in the storage tank was automatically delivered to rice seedlings in the growing container via drip emitter arrays at 6‐h intervals, providing a cumulative daily irrigation volume of 1.2 L to the cultivation assembly.

## Conflict of Interest

The authors declare no conflict of interest.

## Supporting information



Supporting Information

Supplemental Video 1

Supplemental Video 2

Supplemental Video 3

Supplemental Video 4

Supplemental Video 5

Supplemental Video 6

## Data Availability

The data that support the findings of this study are available from the corresponding author upon reasonable request.
