# Supplementary material for: A Robust Solar Evaporator with Hierarchical Architecture for Ultrahigh Efficiency and Large‐Scale Zero‐Liquid‐Discharge Desalination
Source: Adv Sci (Weinh). 2025 Nov 7;13(5):e17735. doi: 10.1002/advs.202517735 (PMC12849996; doi:10.1002/advs.202517735)
Supplement: Supplementary file 1 — Supporting Information [file ADVS-13-e17735-s002.docx]

Supporting Information

Title

A Robust Solar Evaporator with Hierarchical Architecture for Ultrahigh Efficiency and Large-scale Zero-Liquid-Discharge Desalination

Lili Sun*, Ning Wang, Ning Hu, Yongyun Zhao, Libin Zhao, Wenbo Chen

Supporting Information contains:

Note S1

Note S2

Note S3

Supporting Figures 5-33

Supporting Tables 34-37

Supporting References 37-41

**Note S1**

Since the solar irradiation is not uniformly distributed, integrated absorptance based on first-law balance was used to evaluate the solar energy utilization efficiency of the hierarchical evaporator. The integrated absorption takes into account the capture and utilization of energy across the full spectrum, which can more accurately reflect the performance of evaporators under irradiation. The integrated absorptance was calculated by the following formula:

$$\text{α}\text{=}\frac{\int_{\text{250}}^{\text{2500}} \text{I(}\text{λ}\text{)(1−R(}\text{λ}\text{))d}\text{λ}}{\int_{\text{250}}^{\text{2500}} \text{I(}\text{λ}\text{)}\text{d}\text{λ}}\text{×100\%}$$

Where I(λ) and R(λ) denote the solar spectral radiation and the reflectance of the sample at different wavelengths, respectively.

The integrated absorptance, calculated over the solar spectrum from 250 to 2500 nm. By combining experimental reflectance data with theoretical formula, the effective absorptance of the wet evaporator exceeds 92%, indicating strong broadband light harvesting. This high absorptance directly contributes to rapid interfacial heating and sustained vapor generation under solar illumination, even in highly saline environments.

**Note S2 The energy input to the evaporator**

For the 3D evaporators, evaporation area index *EAI* is defined as the radio of the total evaporation surface area to the projected ground area. For the 3D evaporator, the *EAI* increases with height and can be expressed as below Equation:

*EAI* =*A*_total_/*A* _project_ =(*A*_top_ + *A*_side_) / *A*_project_= 1 + (4*h* / *a*) (S1)

where *a* is the length of a side and h is the effective height.

For a given length of a side, a higher *EAI* indicates a larger effective evaporation surface of the evaporator, leading to an increased evaporation rate when standardized to the project area. As *EAI* increases, the system captures more environmental thermal energy, resulting in progressively higher evaporation rates.

The total energy input to the system can be analyzed using the below Equation:

*Q_input_* = *Q_solar_* + *Q_conv_*+*Q_r_*+*Q_water_* (S2)

where *Q*_input_ is the total energy input to the system; *Q*_solar_ represents the absorbed solar irradiation (1.0 kW m^-2^); *Q*_conv_ and *Q*_r_ denotes the heat flows from the surrounding environment via convective and radiative, respectively; *Q*_water_ refers to the heat transfer from the bulk water.

The solar energy input can be determined by equation (S3):

Q*_solar_* = *I_0_A_t_*  (S3)

where *I*_0_ is the incident solar irradiance (1.0 kW m^-2^), and *A_t_* is the top surface area (projected area) of the evaporator.

The convection heat transfer from ambient environment can be quantified by equation (S4-S7):

Q*_conv_* = *h_conv_* [(*A*_s_ (*T_amb_*-*T_side_*)+(*A_t_* (*T_amb_*-*T_top_*)] (S4)

*h*_conv_=*N_uL_ k / L,*  (S5)

*N_ul=_ 1.07 (G_rL_P_r_)^0.28^* (S6)

*G_rL_=g(T_amb_*-T*_side_)L^3^/ T_a_ν²* (S7)

where *hconv* is the convective heat transfer coefficient through air (5 W m^-2^ K^-1^), *T_amb_* is the the ambient temperature above the surface, *T_side_* is the temperature of the evaporator side surface, *A_s_* is the side surface area of the evaporator, *L* is the height of the evaporator, *k* is the thermal conductivity of air, *P_r_* is the Prandtl Number for air, g is the acceleration of gravity (9.8 m s^-2^), and *v* is the kinematic viscosity of air.

The radiation heat transfer from ambient environment can be calculated by Stefan-Boltzmann law:

$Q_{r}=\varepsilon\sigma[A_{s}(T_{side}^{4}-T_{amb}^{4})+A_{t}(T_{top}^{4}-T_{amb}^{4})]$ (S8)

where *ɛ* is the emissive rate (0.97), and *σ* is the Stefan-Boltzmann constant (5.67× 10^-8^ W m^-2^ K^-4^).

The energy transfer from bulk water can be calculated by equation (S9):

$Q_{water}=\frac{\text{C}_{\text{p}}\text{ }\text{m }\text{(}\text{T}_{\text{w}}\text{-}\text{T}_{\text{s}}\text{)}}{\text{t}}$ (S9)

where *C_p_* is the specific heat capacity of 10% saline (3.47 x 10^3^ J kg^-1^ K^-1^), *m* is the mass of the bulk water, *t* is the time for the energy transfer from bulk water to evaporator (1 h), and *T_w_* is the temperature of the bulk water.

**Note S3 The evaporation efficiency of the evaporator**

According to the different states of water molecules in the gel, water can generally be divided into three categories: bound water (BW), intermediate water (IW), and free water (FW). Among them, intermediate water has the lowest evaporation enthalpy, which is most conducive to improving energy efficiency. The hydrogen bond interaction between the mycelial molecular chains and water molecules promotes the generation of a large amount of IW and some BW in the system, thereby reducing the overall evaporation enthalpy of water and enhancing the photothermal conversion efficiency.

The same surface area of bulk water, pure water, 5, 10, 25 wt.% NaCl in evaporator were synchronously placed in a closed container together with a supersaturated magnesium chloride solution (which enabled a stable RH of 33%) at 25 ºC and ambient air pressure. The distances between the samples were the same. Mass losses were recorded after 90 mins. The equivalent evaporation enthalpy (Δ𝐻_𝑒𝑞𝑢_) of bulk water, evaporator with different concentrations of brine were calculated by vaporizing the water with identical power input (U_in_) :

𝑈_𝑖𝑛_ = Δ𝐻_0_𝑚_0_ = Δ𝐻_𝑒𝑞𝑢_𝑚_𝑔_  (S10)

where Δ𝐻_0_ and 𝑚_0_ are the evaporation enthalpy (2442 kJ kg^–1^) and mass loss of bulk water; *m*_g_ is the mass loss of evaporator. The corresponding calculated Δ𝐻_𝑒𝑞𝑢_ was shown in Figure S20, showing that the evaporation enthalpy of the evaporator in pure water were 630, 1083, 1165, 1015 J g^-1^,respectively, significantly lower than the bulk water (2442 J g^-1^).

Based on the results of dark evaporation experiments, the photothermal conversion efficiency (*η*) of the evaporator is calculated using the following equation:

*η* = *Ṁ* (*C_p_ ΔT*+Δ𝐻) / (*C*_opt_*I*) (S11)

*Ṁ* = *M_e_*-*M_d_* (S12)

where *Ṁ* is the net evaporation rate of evaporator at a steady state, kg m^-2^ h^-1^; *C_p_* is the specific heat capacity of water (4.18 kJ kg^-1^ K^-1^); *Δ T* is temperature difference between the vapor and the environment, ℃; Δ𝐻 is the equivalent vaporization enthalpy of the water, kJ kg^-1^; *C*_opt_ refers to the optical concentration on the absorber surface; *I* is the solar irradiation power, kW m^-2^; and *M_e_ and M_d_* are the evaporation rates of evaporators at a steady state under 1 sun and dark condition ( kg m^-2^ h^-1^), respectively.

According to this equation, the evaporation efficiencies for the evaporator significantly exceeded the theoretical limit, reaching value of 241% for the evaporators with EAI of 11. This enhancement is primarily attributed to the additional energy gained and increased EAI.

**Supporting Figures**


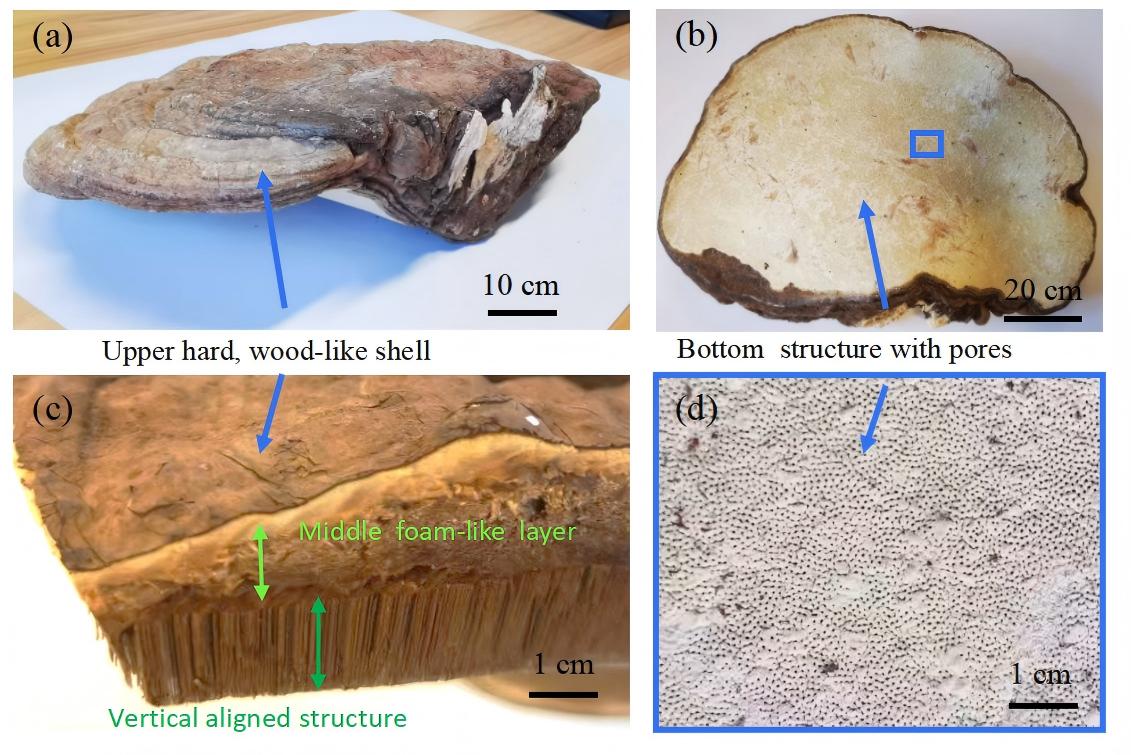


**Figure S1.** The fruit body of *Bracket fungi* and their morphological features. (a) Overall appearance of *Bracket fungi*. (b) Bottom surface. (c) Longitudinal cross-section. (d) The enlarged image in (b).


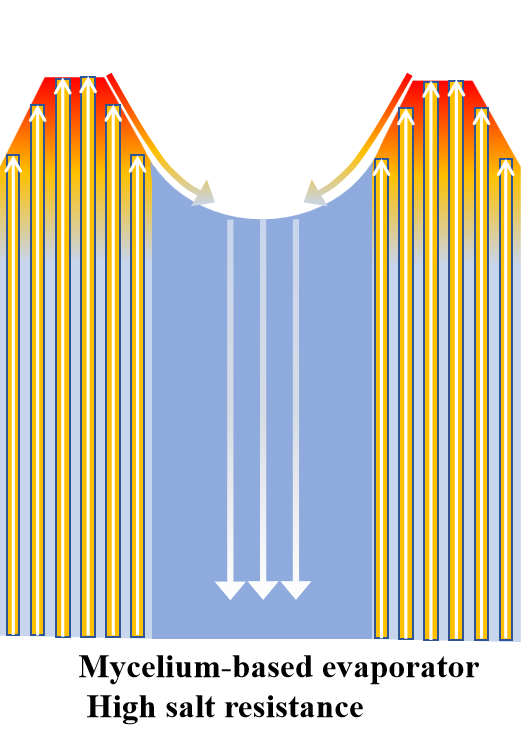


**Figure S2.** Schematic illustration the directional water transport through internal fungal channels, and the concentrated brine flows into the tube channel of mycelium-based evaporator. As for the state-of-the-art evaporators,^[1]^ it is difficult to realize the spatial isolation of concentrated salt between water transport area and evaporation area.

***High salt resistance*** While surface modification in most evaporators (such as Janus structure) temporarily boosts evaporation efficiency, its limited water transport performance causes rapid salt accumulation on the surface, making sustained operation difficult. In addition, it is difficult for most evaporators to realize the spatial isolation of concentrated salt between water transport area and evaporation area (Figure S2, a). Our design addresses this by implementing directional water transport through internal fungal channels. These channels are spatially isolated from evaporation zones, preventing concentrated brine reflux into capillary tubes after evaporation. This innovative approach effectively avoids water channel blockages and ensures long-term stable water transfer (Figure S2,b).

The inclined trapezoidal surface creates a crescent-shaped water level in the tube hole that remains below the evaporation surface. This configuration prevents excessive water gathering at the evaporation front, while the lower water level ensures the lower temperature compared to the evaporation surface. The thermal gradient generates surface tension gradients, known as the Marangoni convection effect. Under the combined influence of Marangoni convection and gravity, concentrated brine flows along the trapezoidal surface into the tube channel and downward along the tube channel under gravity.


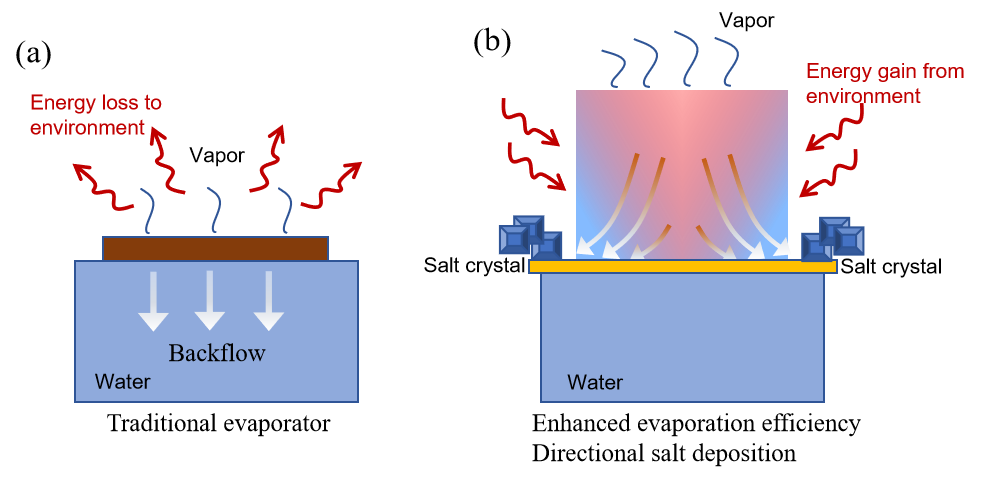


**Figure S3.** (a) Schematic illustration of the evaporation process for the traditional evaporators. (b) Schematic illustration of the enhanced evaporation efficiency and the directional salt deposition for the mycelium-based evaporator.

***Directional salt collection***: The evaporator's side surface functions as a cooling surface. Through evaporation cooling, the temperature becomes lower than both ambient and central evaporator temperatures. This creates a temperature gradient from the core to the periphery within the evaporator. Meanwhile, the salt concentration on the side surface exceeds that in the central region, forming a solute concentration gradient. This configuration further enhances the Marangoni convection effect, driving the recirculating brine to migrate directionally toward the bottom sidewall.

***High evaporation efficiency***: Under the thermal gradient, the side of the evaporator can absorb heat from the surrounding environment through convection and radiation, which can not only improve the evaporation efficiency, but also effectively compensate for the heat loss of the top surface, realizing the self-circulation of energy. Compared to most of the evaporators, the additional energy gained and increased EAI make the evaporation efficiency higher than the theoretical limit (Figure S3).


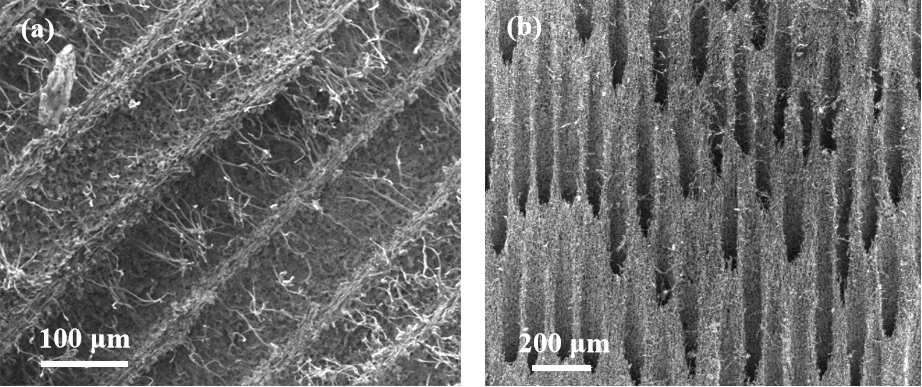


**Figure S4.** Microstructure characterization of the hierarchical mycelium. The longitudinal sectional (a) and the oblique sectional (b) SEM images of the the middle mycelium, confirming the vertical tubular channels within the fungal skeleton..


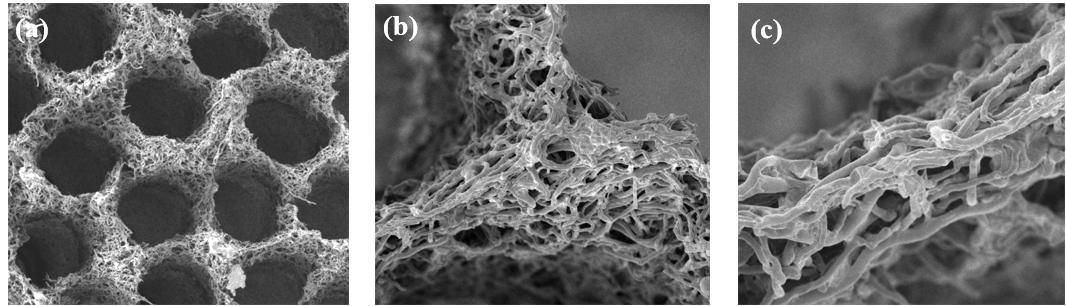


**Figure S5.** Microstructure characterization of the hierarchical mycelium. (a) Top-view SEM image of the mycelium. Partial magnified SEM image in (a) showing (b) the Fungal mycelium skeleton at the junction of the tube. (c) The partial magnified SEM image in (b) showing the network of hyphae arranged in a horizontal order at the top.

**
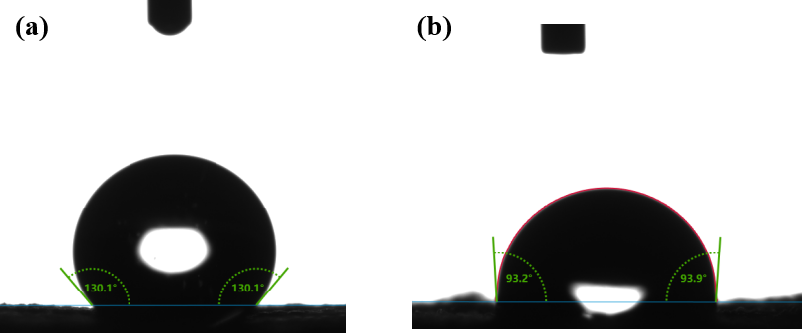
**

**Figure S6.** Contact angle images of evaporator under 10 wt.% brine before and after 72 h light irradiation.


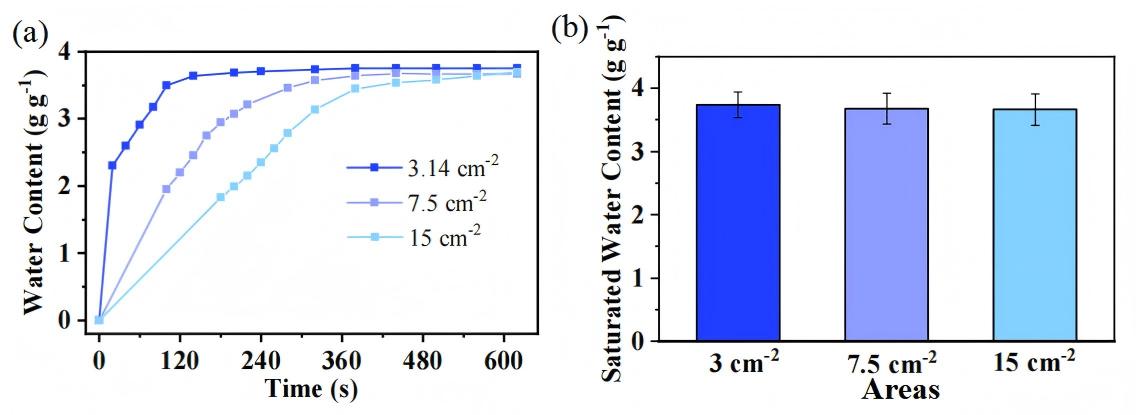


**Figure S7.** Water transportation of the evaporators. (a) Water content variation over time for evaporators with varying areas. (b) The saturated water content in evaporators with varying areas.


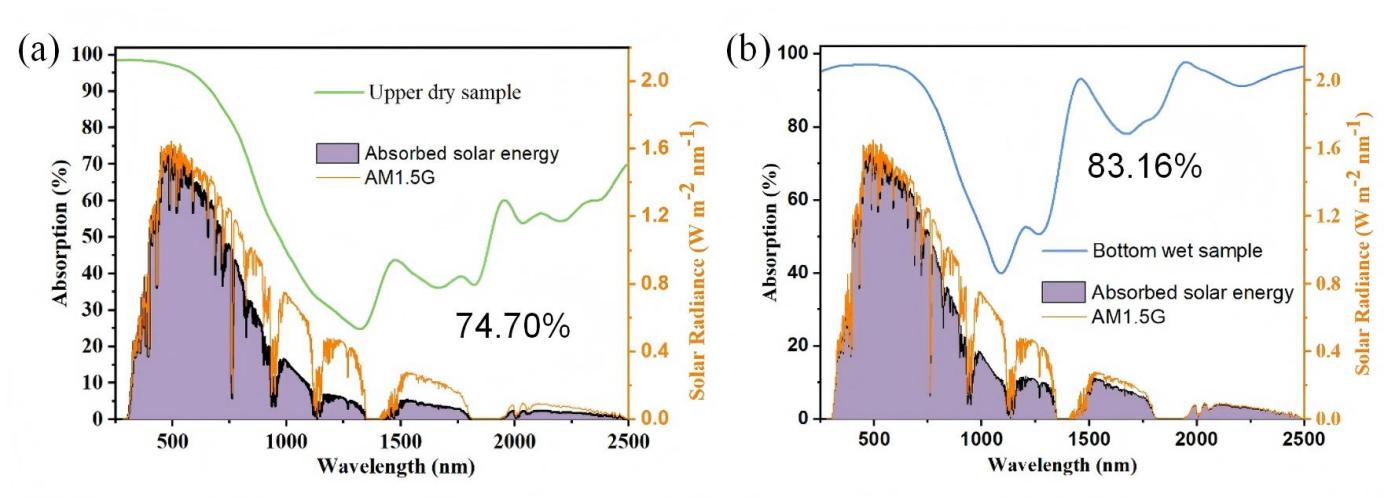


**Figure S8.** Photothermal performance of the evaporators. The UV-Vis-NIR spectra of (a) the upper surface of the dry sample, and (b) the bottom surface of the wet sample.


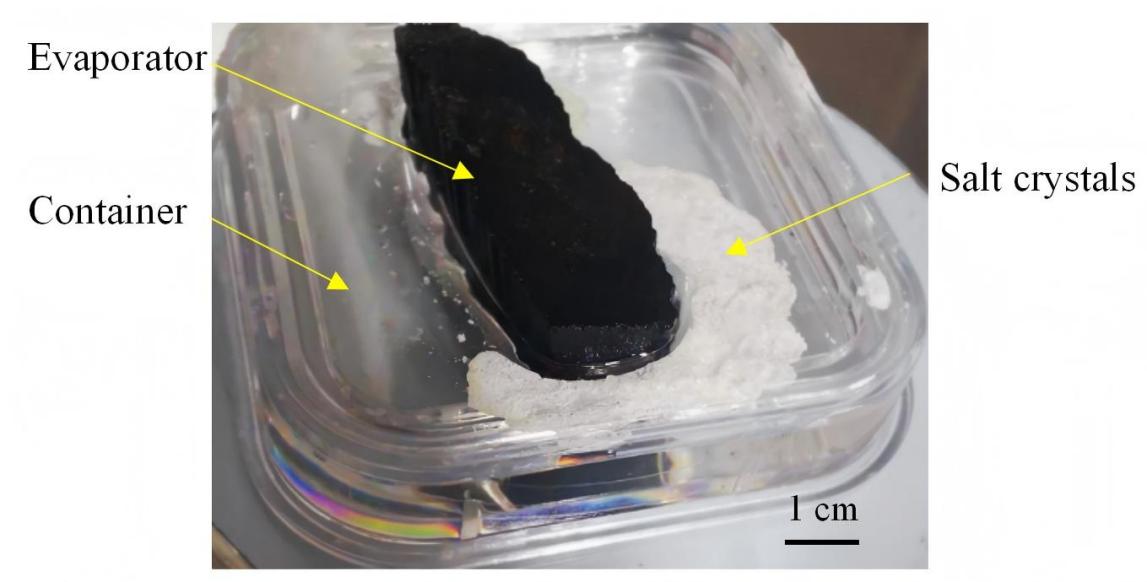


**Figure S9.** Photograph of the evaporator device. The salt crystals deposited at the bottom edge of the evaporator.


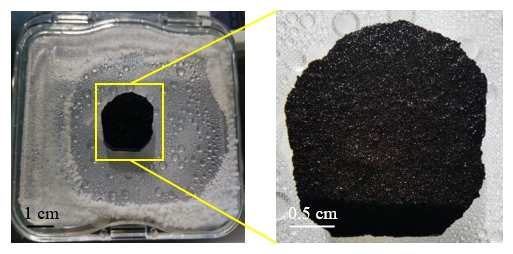


**Figure S10.** Photograph of the evaporator device after continuous 12 h ZLD desalination process, no salt accumulation occurred on the evaporator surface.


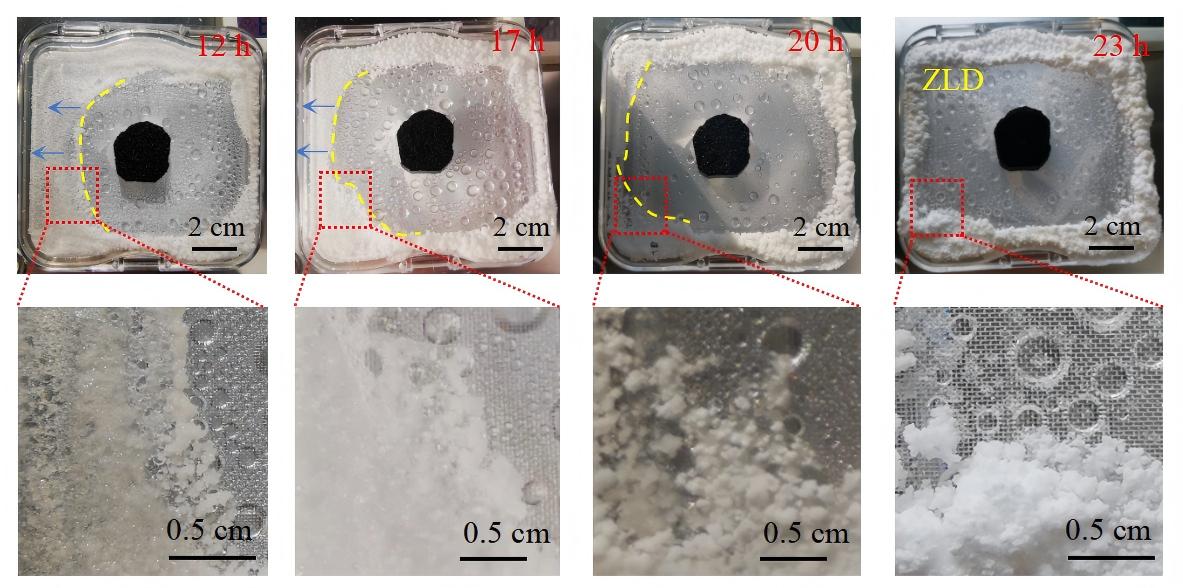


**Figure S11.** Photographs of the evaporation device during desalination test under the natural sunlight. The enlarged image shows that the salt crystals were loose and porous during the desalination process.


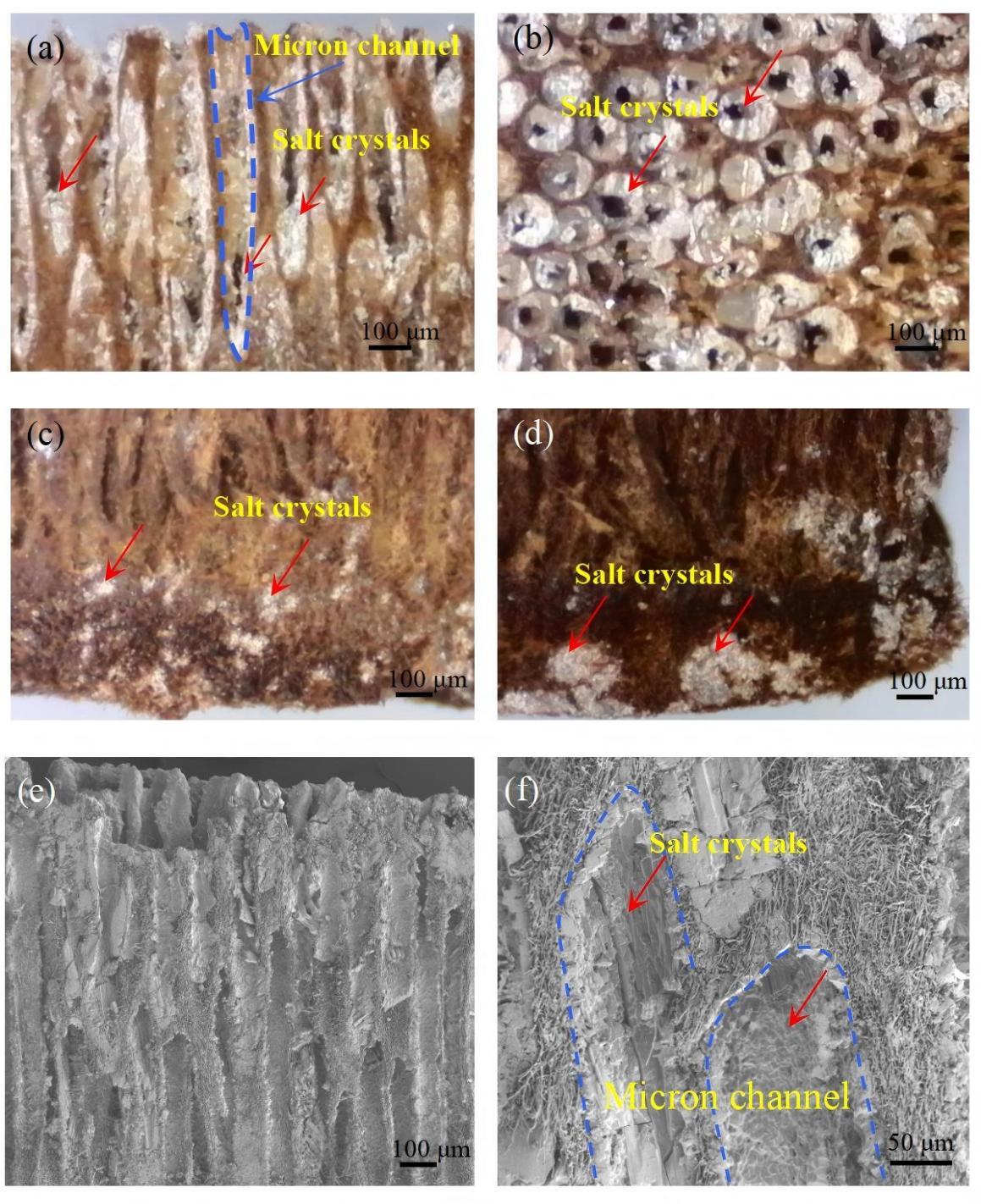


**Figure S12.** The longitudinal sectional (a, c, d) and the cross-sectional (b) optical microscope images of the mycelium employed for desalination, showing the salt crystals deposited within the micron channels and the bottom interstices of the hyphae network. (e, f) The longitudinal sectional SEM images, distinctly reveal the deposited salt crystals within the micron channels, while the hyphae skeleton remains porous structure.


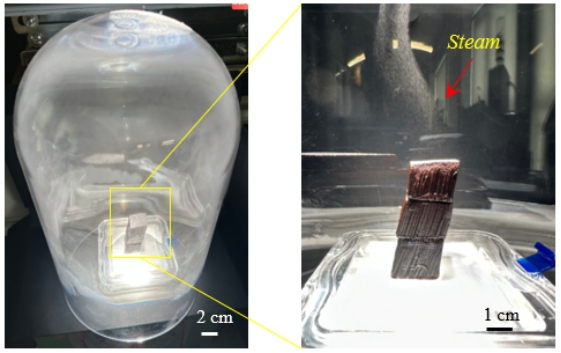


**Figure S13.** Photographs of the evaporation-condensation device under 4 sun illumination. The steam is clearly observed escaping continuously from the evaporator surface.


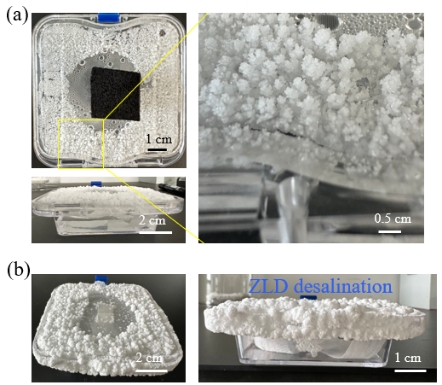


**Figure S14.** (a) Photographs of the evaporation device during the desalination test. The enlarged image shows the wetted dendritic salt crystals, which enhance the evaporation rate by the augmented interfacial evaporation areas. (b) Photographs of the evaporation device at the end of ZLD desalination.


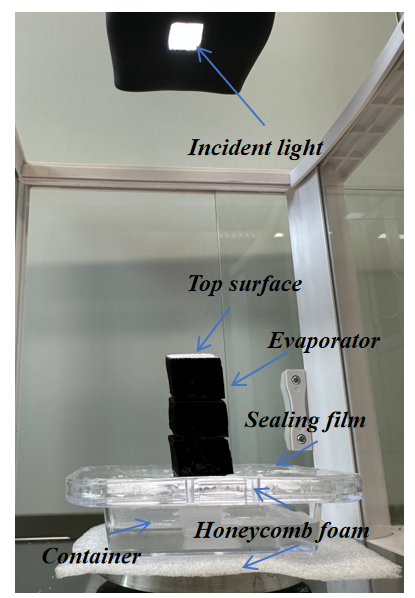


**Figure S15.** Illustration of experimental setup during the vapor generation test. The surface of the container is covered with sealing film to prevent direct evaporation of the underlying bulk brine from affecting the experimental results. And a honeycomb foam is placed beneath the sealing film to avoid the additional heating to the water body. In addition, the size of the light spot was controlled by an aperture, ensuring that the illumination area matched exactly the projected area of the evaporator.


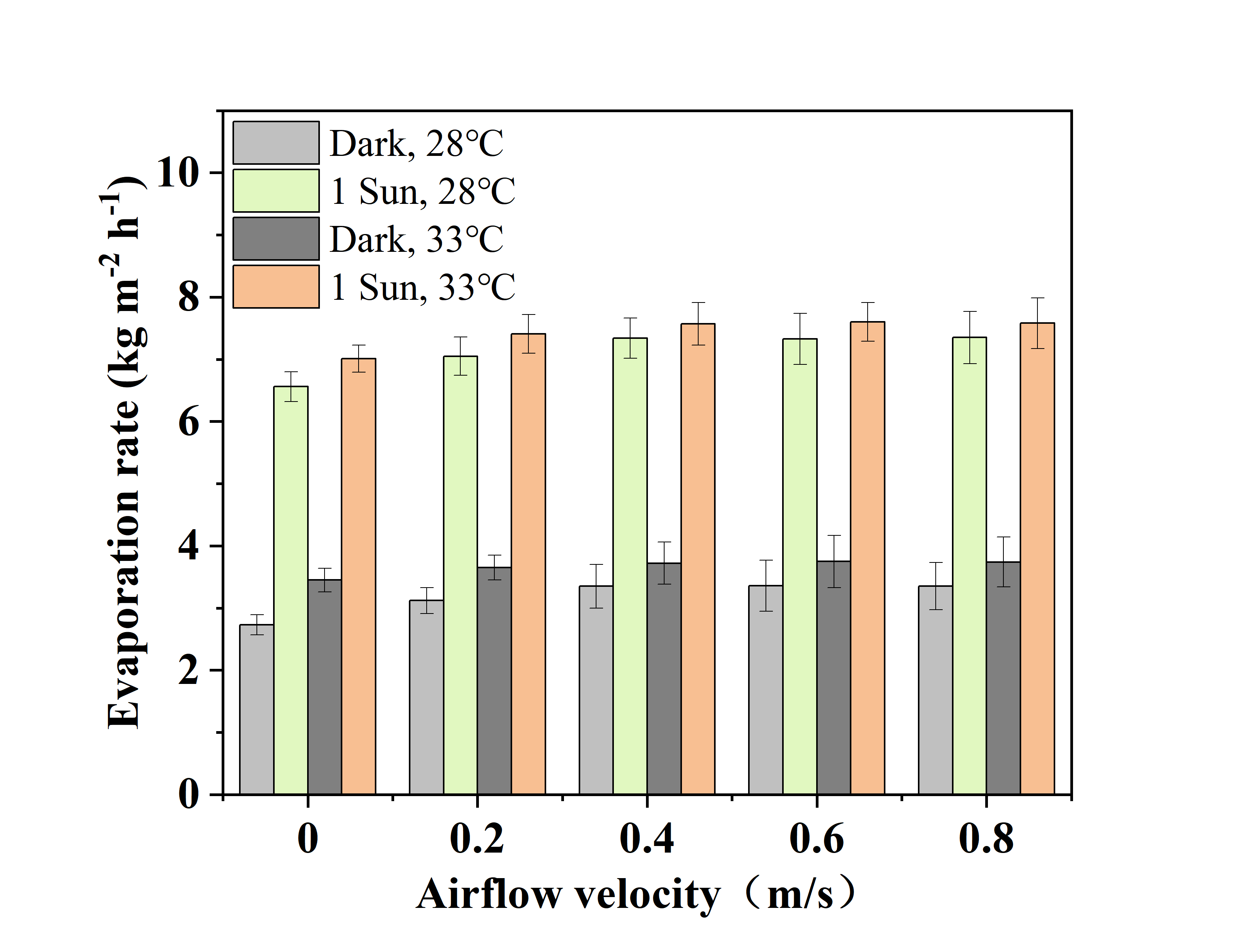


**Figure S16.** Evaporation rates of 10 wt.% brine with airflow and temperature changes for evaporators with height of 5 cm and area of 4 cm^2^ under 1 sun illumination and dark, relative humidity is 50%. Airflow velocity was regulated using a precision anemometer (FLUKE, F923), allowing for consistent and repeatable measurements. All data were recorded over a period of 120 minutes to ensure steady-state evaporation was achieved. Each measurement was repeated three times to confirm reproducibility, with error bars representing standard deviations. The evaporation rates exhibited an enhancement on airflow velocity, with higher rates observed under 33 ℃ compared to 28 ℃, both in 1 sun illumination and dark conditions. The evaporator achieved a maximum rate of 7.57 kg m⁻² h⁻¹ at 33 ℃ and 0.8 m/s airflow, demonstrating synergistic enhancement from convective mass transfer.


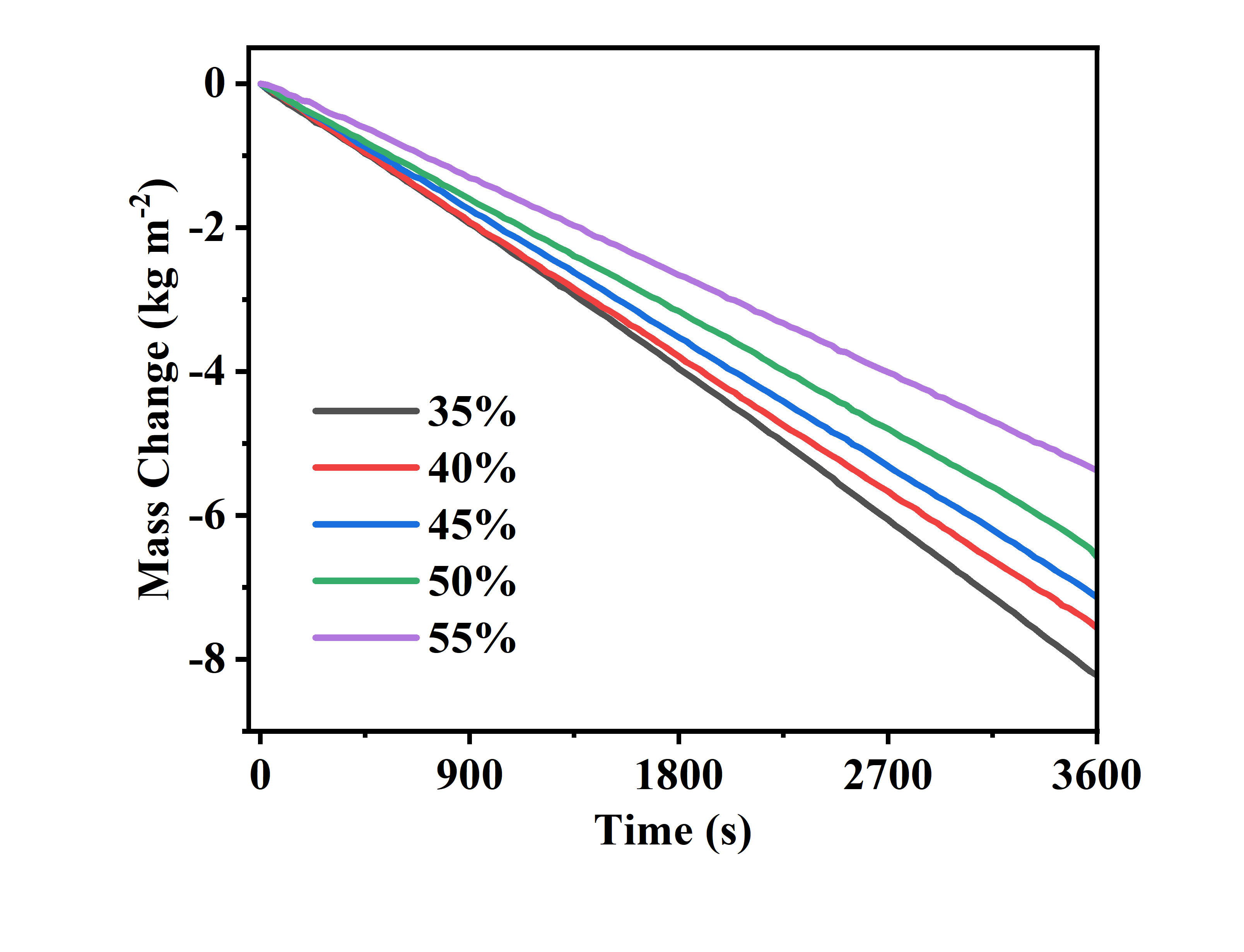


**Figure S17.** Mass change curves of 10 wt.% brine with humidity changes for evaporators with height of 5 cm and area of 4 cm^2^ under 1 sun illumination.


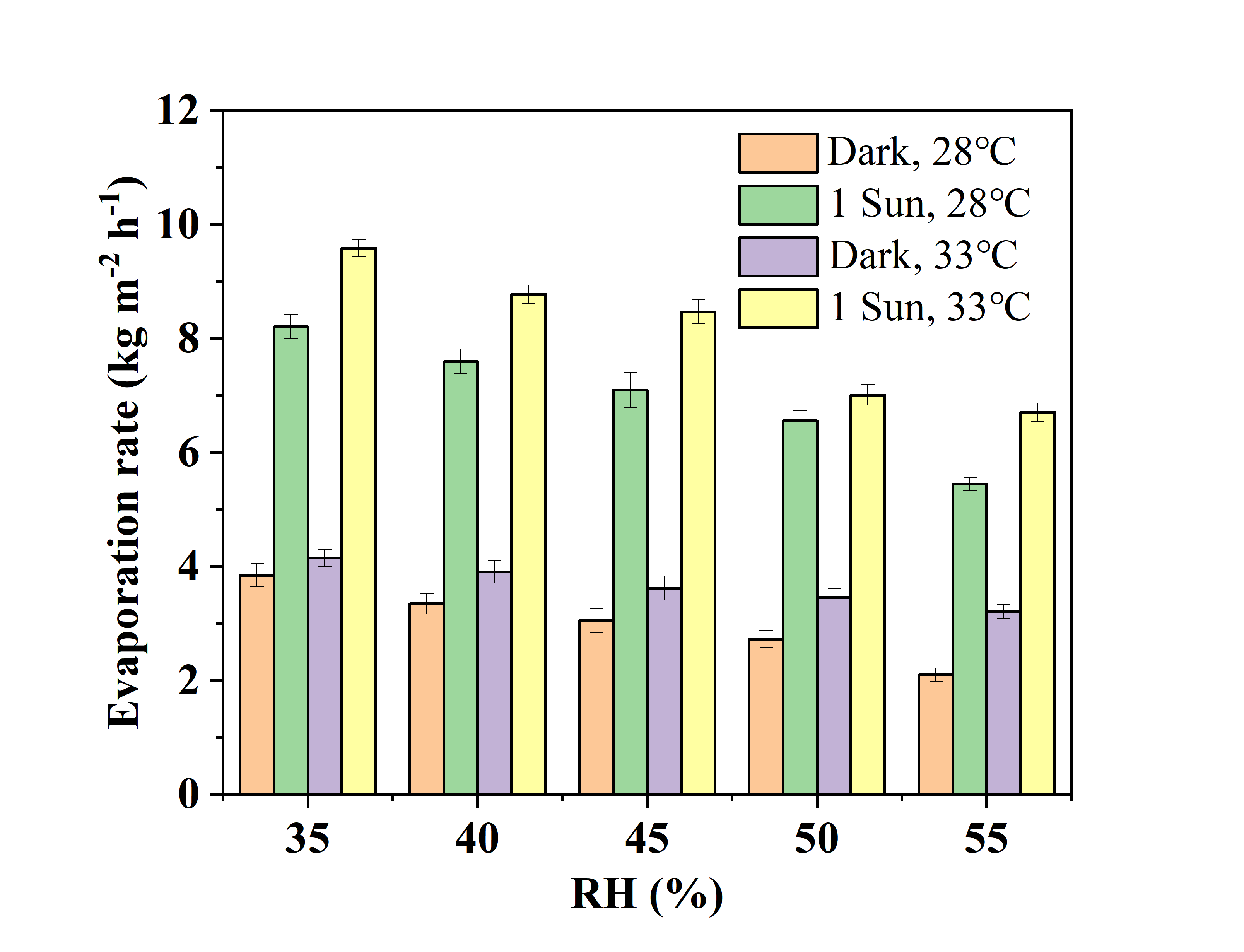


**Figure S18.** Evaporation rates of 10 wt.% brine with relative humidity and temperature changes for evaporators with height of 5 cm and area of 4 cm^2^ under 1 sun illumination and dark, airflow velocity is 0 m/s. Data from Figure S11 and S12 indicate that under 1 sun illumination and in darkness, the rate decreases with increasing relative humidity, consistent with the principle that saturated air impedes further water vapor diffusion.


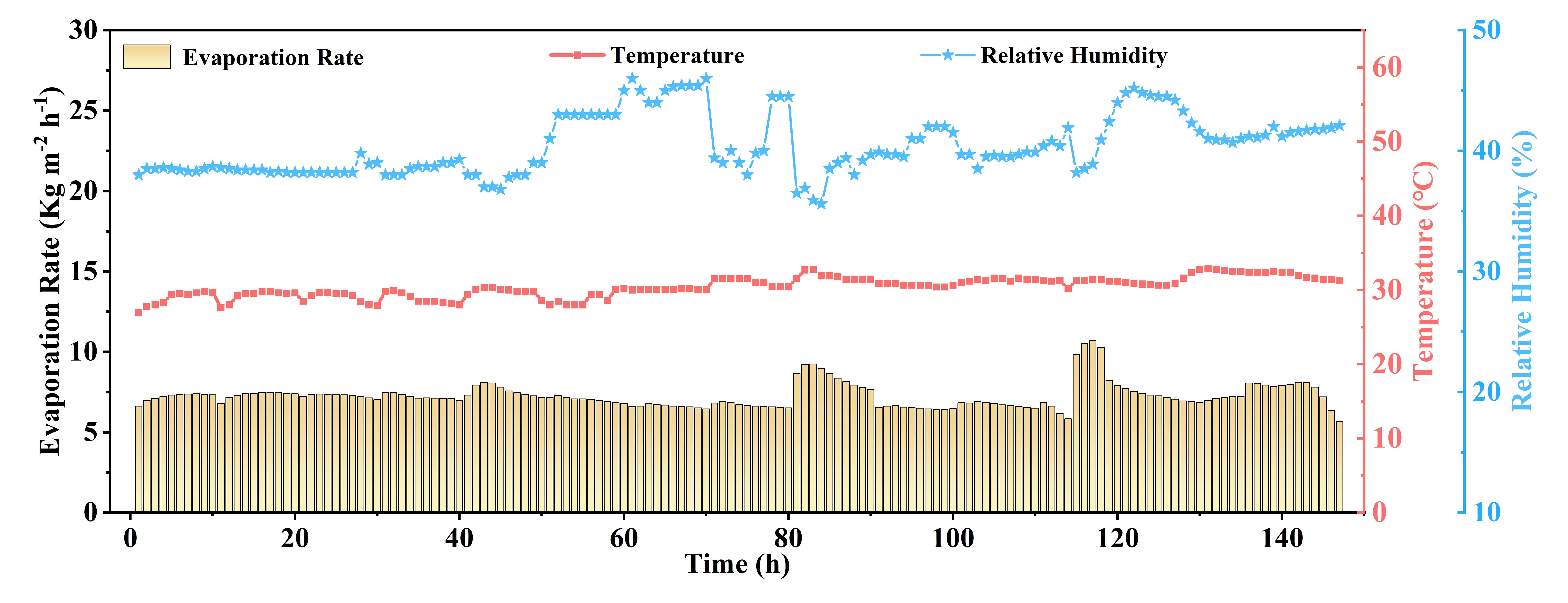


**Figure S19.** Evaporation rates and environmental conditions during the four cycles of ZLD desalination test.


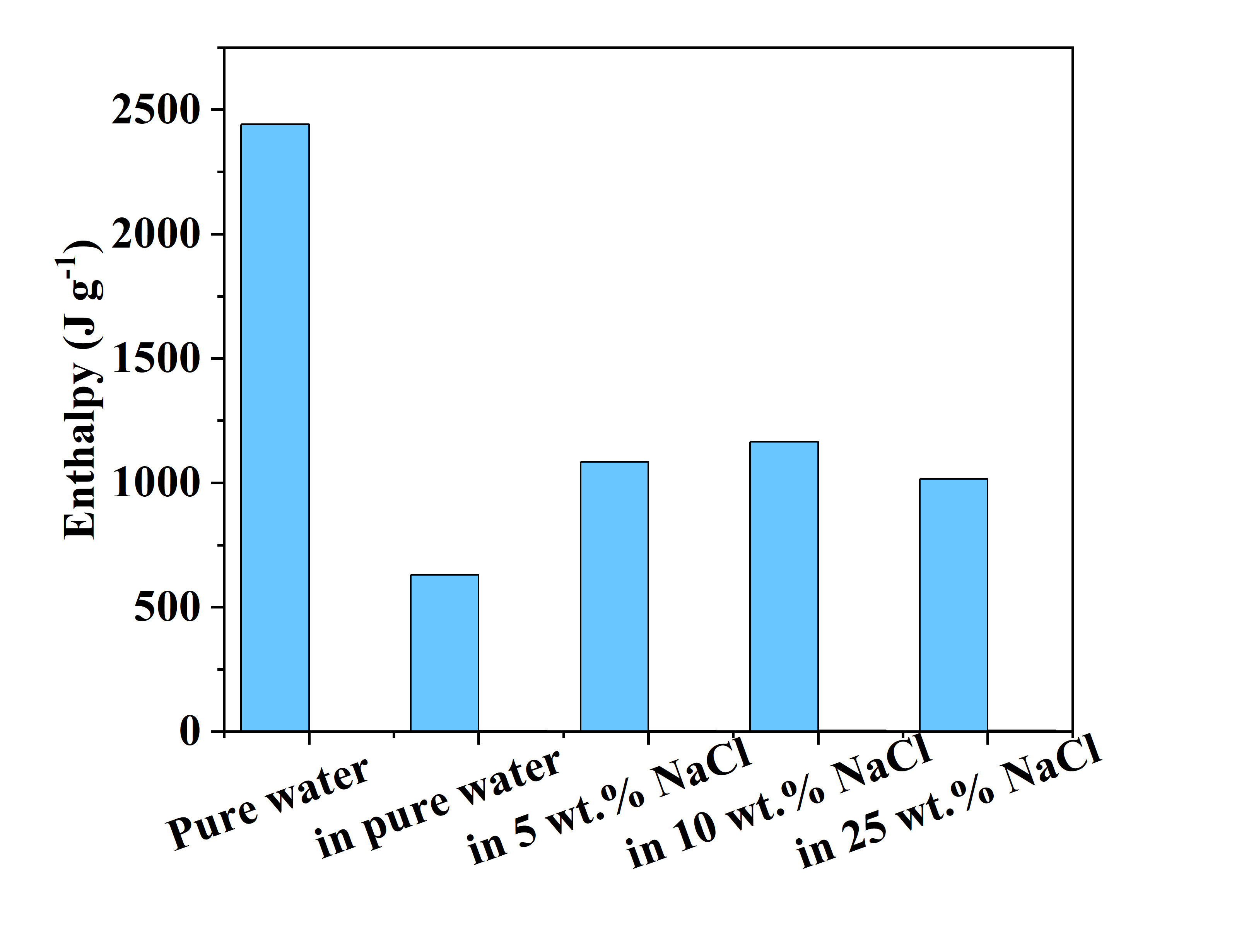


**Figure S20.**  Equivalent vaporization enthalpy of the pure water and the evaporators in pure water, and 5, 10, 25 wt.% NaCl.


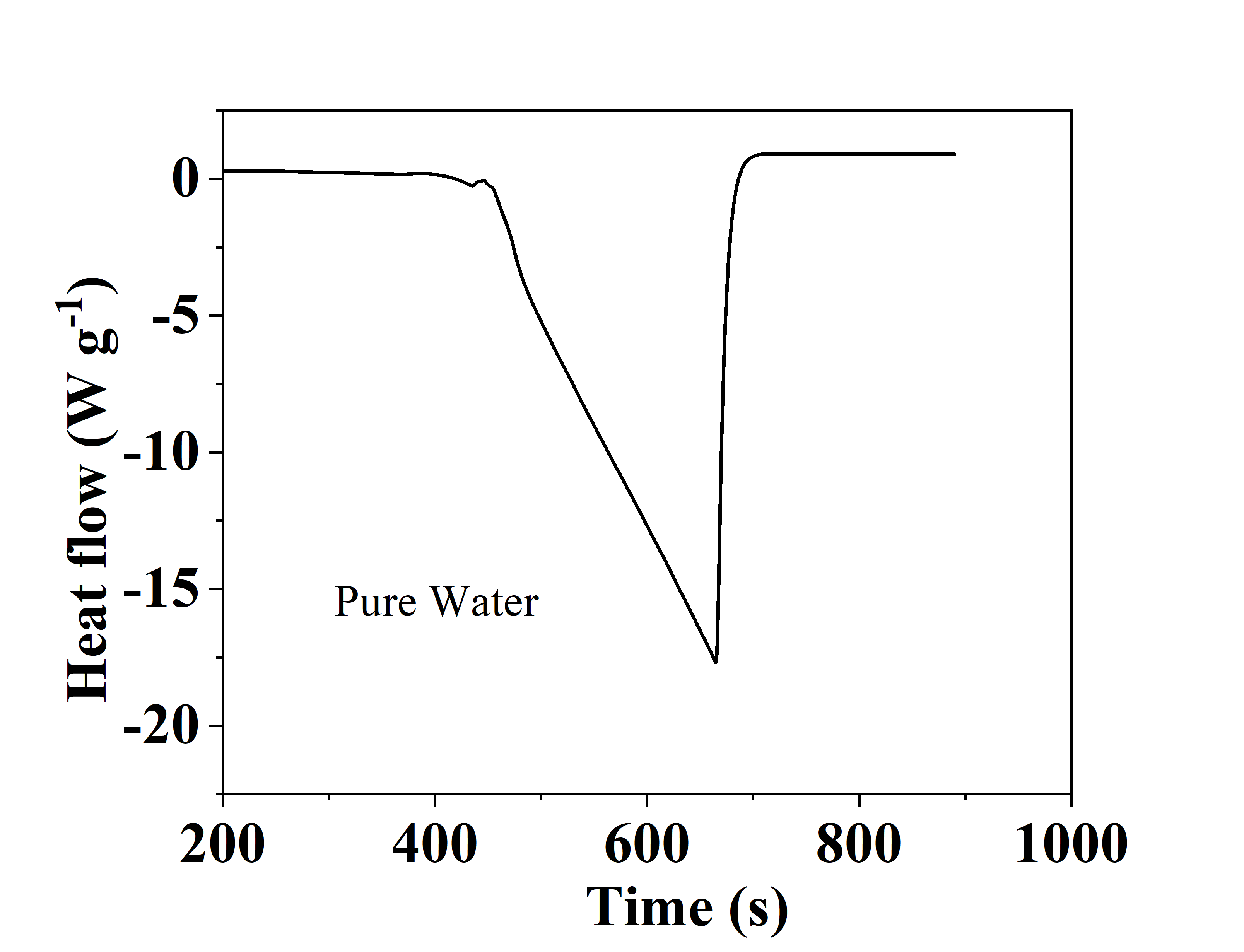


**Figure S21.**  DSC curves of the pure water.


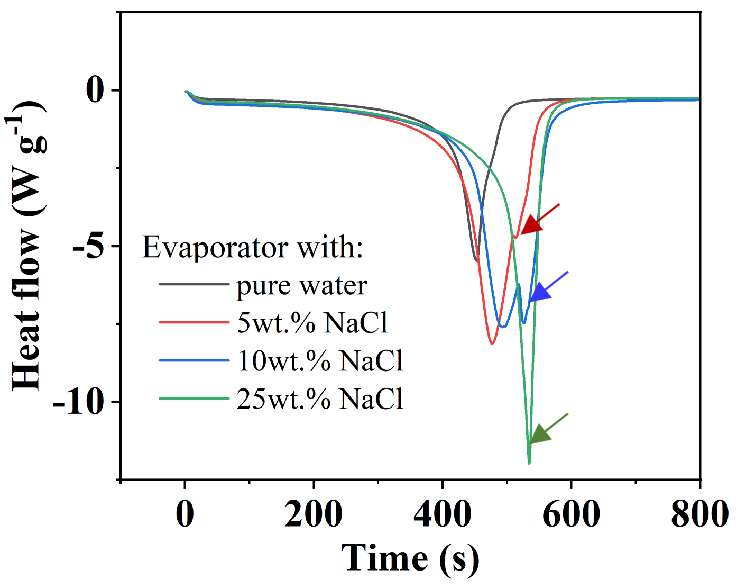


**Figure S22.** DSC curves of the evaporator in pure water, 5 wt.%, 10 wt.%, and 25 wt.% NaCl.

A sharp peak and a rapid decay after the signal reached a maximum occurred, indicating a different evaporation behavior compared to the low concentration of brine. The reduced evaporation enthalpy in the higher salinity is primarily attributed to the formation of abundant IW. At 5 wt.% NaCl, a weaker peak begins to appear, as indicated by the red arrow. And then the peak value continuous to grow as the salt concentration. Considering the lower energy barrier of the IW and the decreased vaporization enthalpy of the saturated brine, it can be inferred that reconstruction of hydrogen bond network within the biomolecule by high concentration ion to water molecule plays an important role in reducing the evaporation enthalpy.


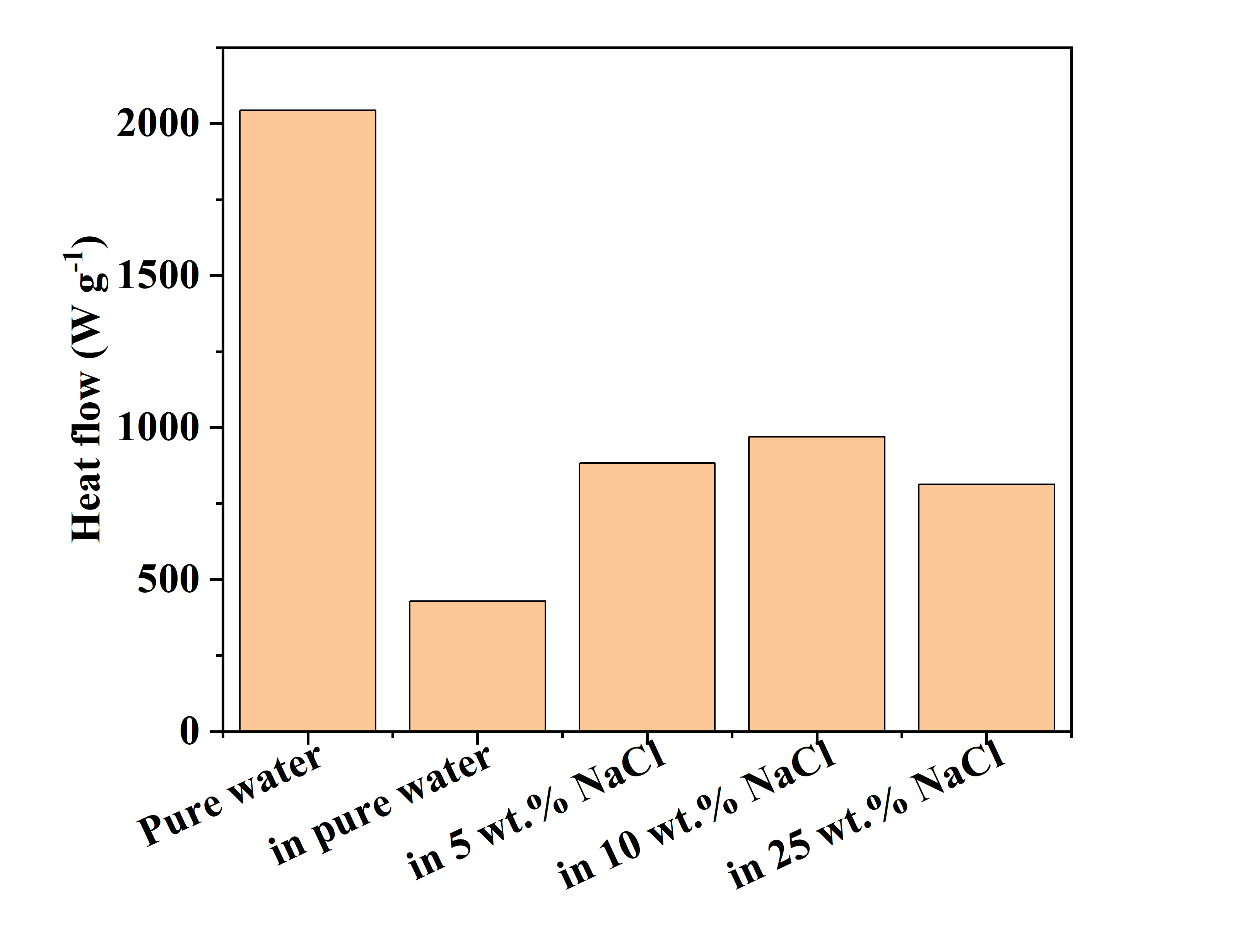


**Figure S23** Evaporation enthalpy of the pure water and the evaporators in pure water, and 5, 10, 25 wt.% NaCl based on DSC analysis. The DSC results showed the lower evaporation enthalpy in the evaporator compared to pure water (2043 J g^-1^), even in brine. The result is consistent with the dark experiments, suggesting that the hydrogen bond interaction between the mycelial molecular chains and water molecules promotes the generation of the IW and some BW in the system, thereby reducing the evaporation enthalpy. In addition, the evaporation enthalpy of the evaporator in the pure water is lower than that in the brine, suggesting that the brine increases the latent heat of vaporization. However, as the salt concentration increase to saturation, the vaporization enthalpy decreased to 813 J g^-1^ instead.


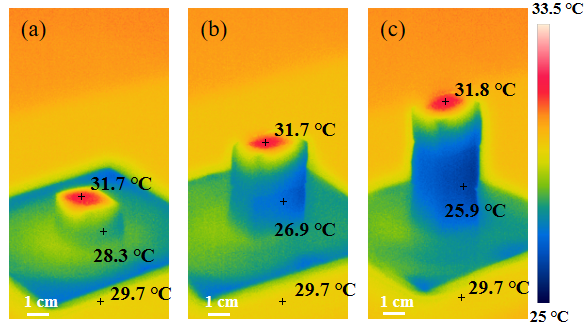


**Figure S24.**  IR thermal images showing the side and top surface temperature of the evaporator with heights of 2.4, 4, and 5 cm under one sun.


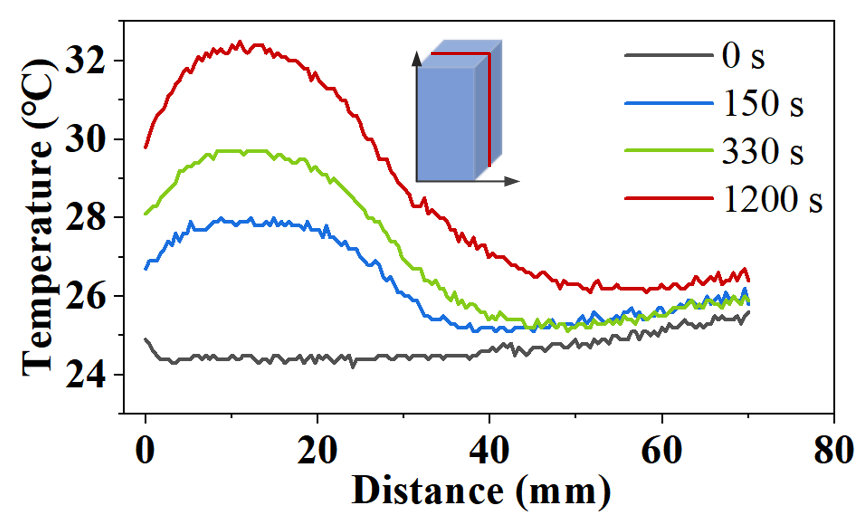


**Figure S25.** The temperature distributions along the red line on the evaporator.


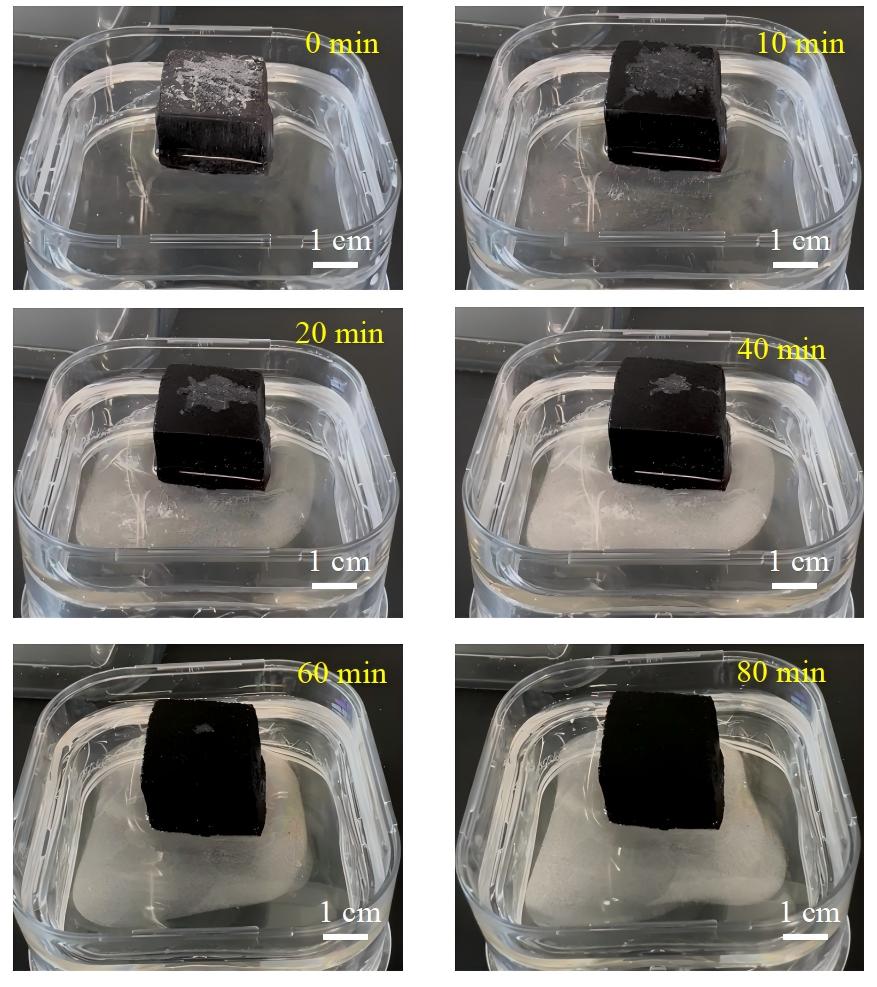


**Figure S26.** Photographs of the evaporator immersed in the water after ZLD desalination test. The residual salt crystals within the evaporator at the end of the ZLD solar evaporation were dissolved within 80 min, confirming the excellent water transfer and salt resistance.


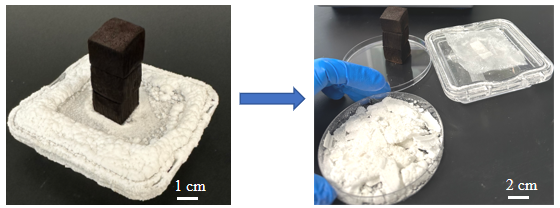


**Figure S27.** The photograph shows the weight of the collected salt after four cycles of ZLD desalination test. The total mass of the NaCl used in the four cycles is m_1_. Before the test, the evaporator device and the evaporator were dried and measured, the weight of the evaporator device and the evaporator was recorded as m_2_. After four cycles of ZLD desalination, the evaporator device and the evaporator were dried and weighed again. The salt crystallization is mainly concentrated on the surface of nylon mesh and the edge of the container, which is evenly distributed and easy to peel. Then the salt crystals were carefully collected and weighed, yielding a total salt mass m_3_. The final weight of the evaporator device and the evaporator after salt removal was recorded as m_4_. The closed NaCl mass balances can be obtained according to the following mass balance formula,

m_1_=m_3_ + m_4_ - m_2_

The salt collection rate *η* can be calculated by the following formula,

*η* = m_3_/ m_1_×100%

We conducted three identical cycles of tests and the weighing results and the slat collection rate are shown in the following table 1.

Table 1 Salt collection efficiency of ZLD

| Number | m_1_ (g) | m_2_ (g) | m_3_ (g) | m_4_ (g) | η (%) |
| --- | --- | --- | --- | --- | --- |
| 1 | 50 | 47.97 | 48.85 | 49.10 | 97.7 |
| 2 | 50 | 47.97 | 48.79 | 49.16 | 97.5 |
| 3 | 50 | 47.97 | 48.80 | 48.14 | 97.6 |

Experimental data show that the measured values of m_1_, m_2_, m_3_ and m_4_ are highly consistent in the three repeated tests, indicating that the operation of the evaporation device is stable, the salt collection process has good repeatability and reliability, the salt recovery rate is kept above 97.5%, which verifies the accuracy and high efficiency of the closed quality balance of the system.


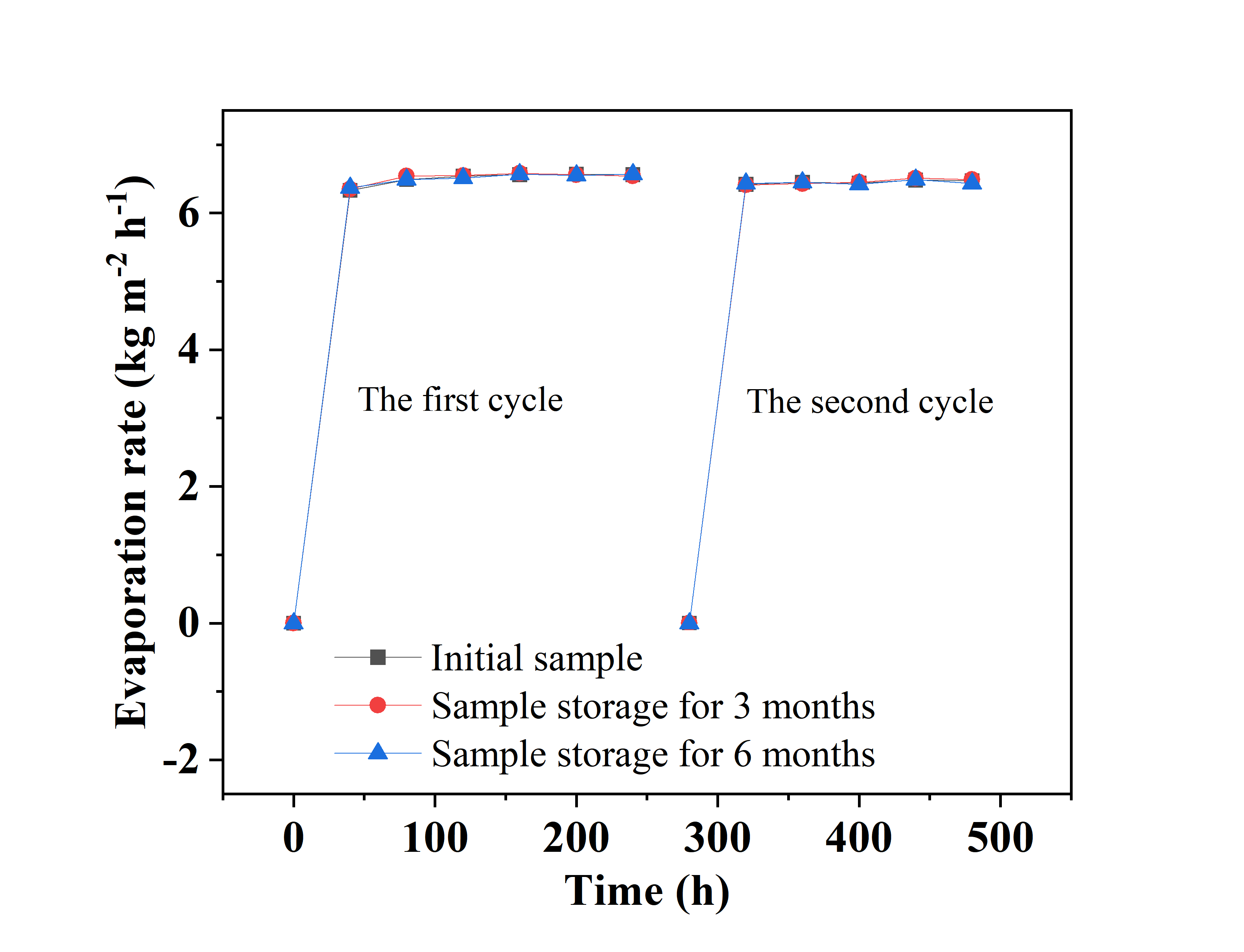


**Figure S28.** The evaporation performance of the evaporators (height=5 cm, area=4 cm^2^) after storage for a long time. The devices maintained high evaporation rates after six months of storage under ambient conditions, demonstrating exceptional structural stability. No significant performance degradation was observed, indicating robust material integrity and sustained functionality for long-term practical.


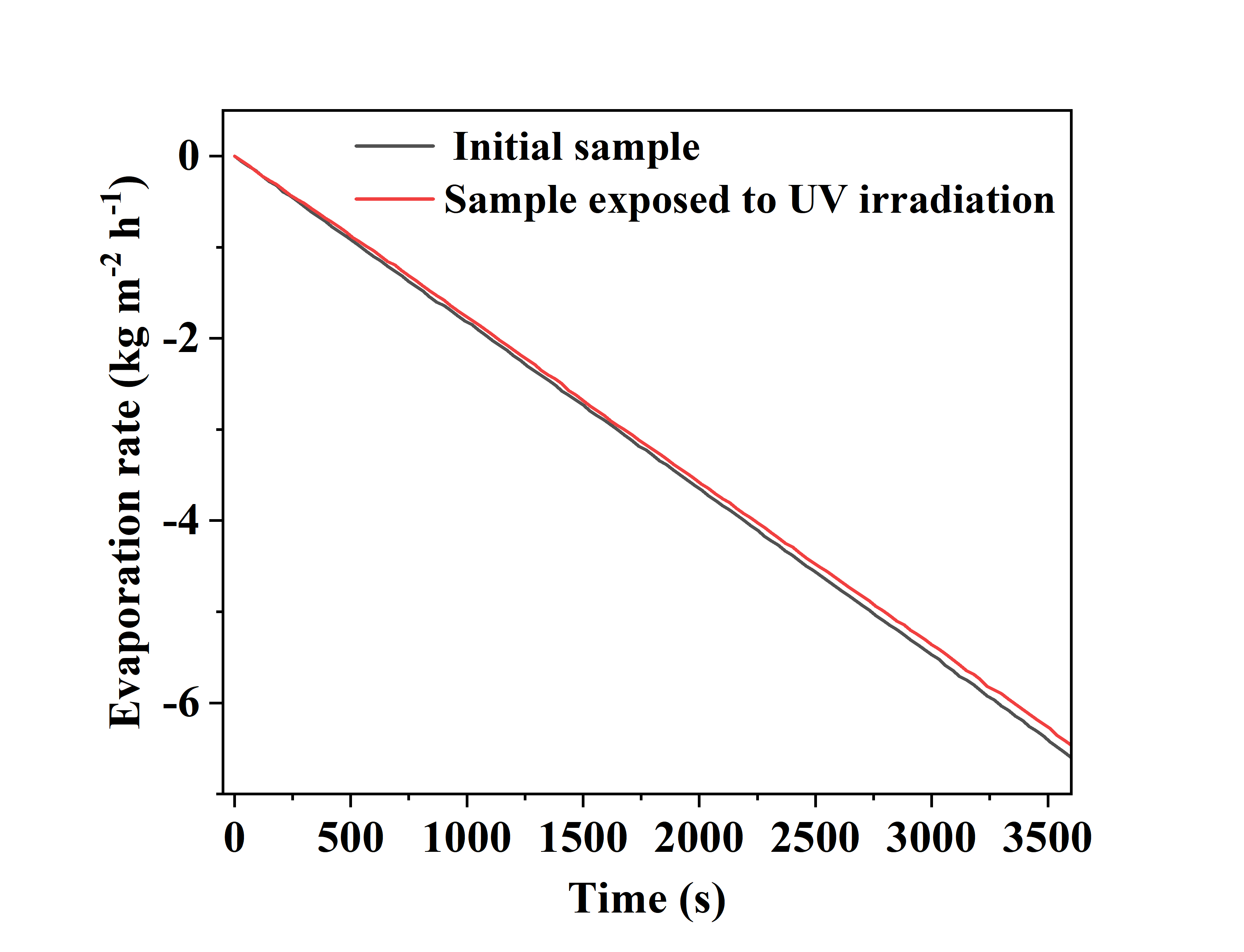


**Figure S29.** The evaporation performance of the evaporators after UV irradiation treatment for 96 hours, showing no obvious decline in evaporation efficiency.


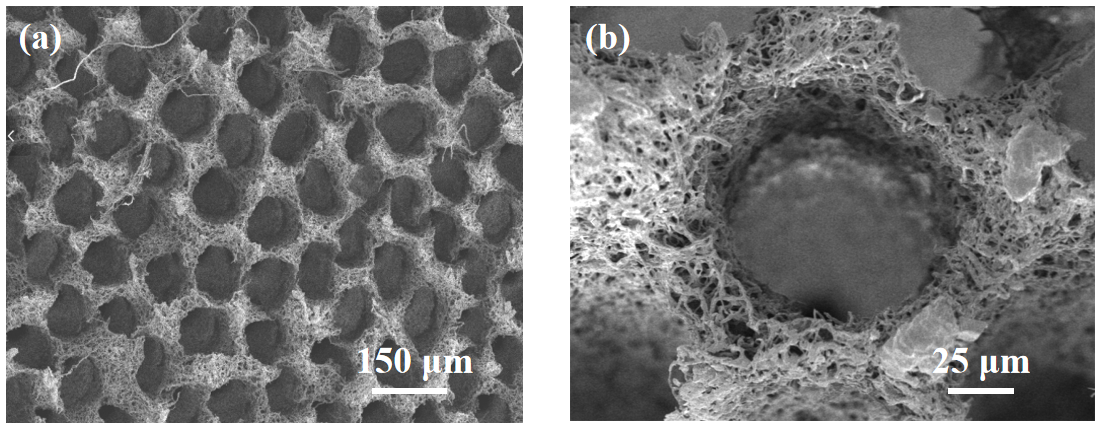


**Figure S30.** SEM images of the evaporator after 6 months storage and UV irradiation treatment for 96 hours. The microstructure of the evaporator remained intact without cracking or delamination, further confirming material's excellent fatigue resistance and durability.


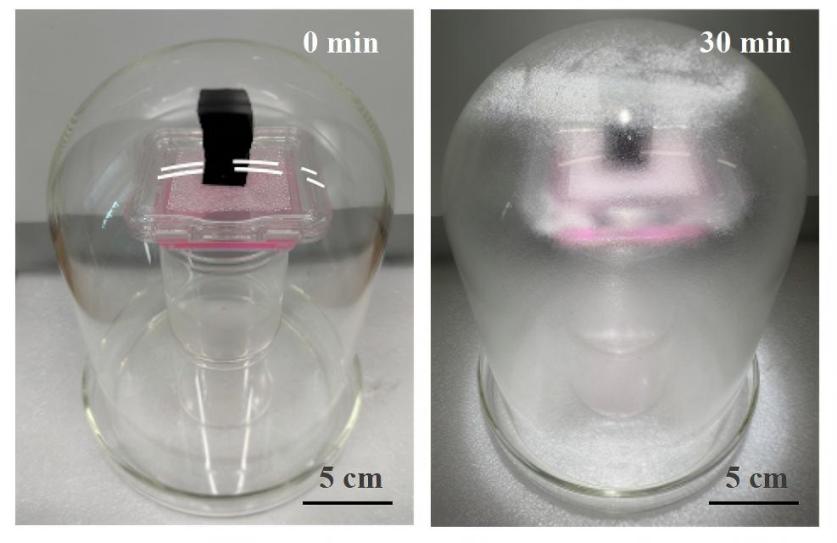


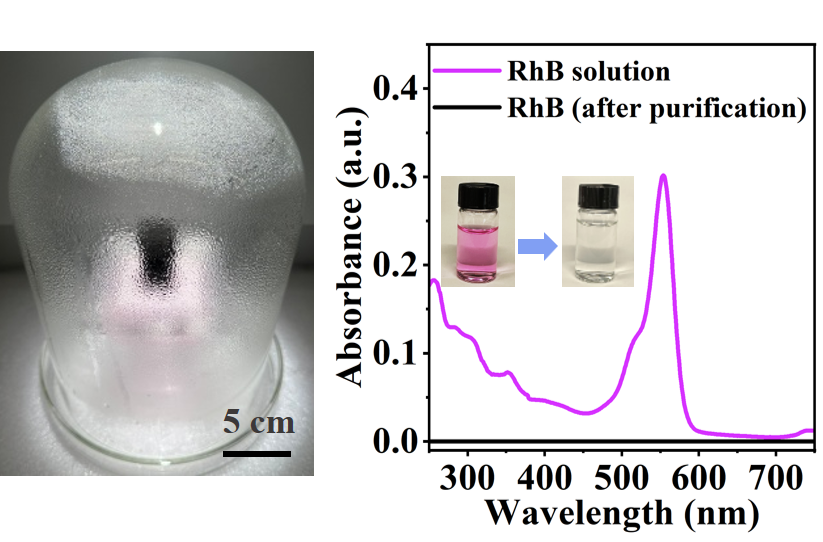


**Figure S31.** The absorption spectral curves of the RhB solution before and after purifying.

**
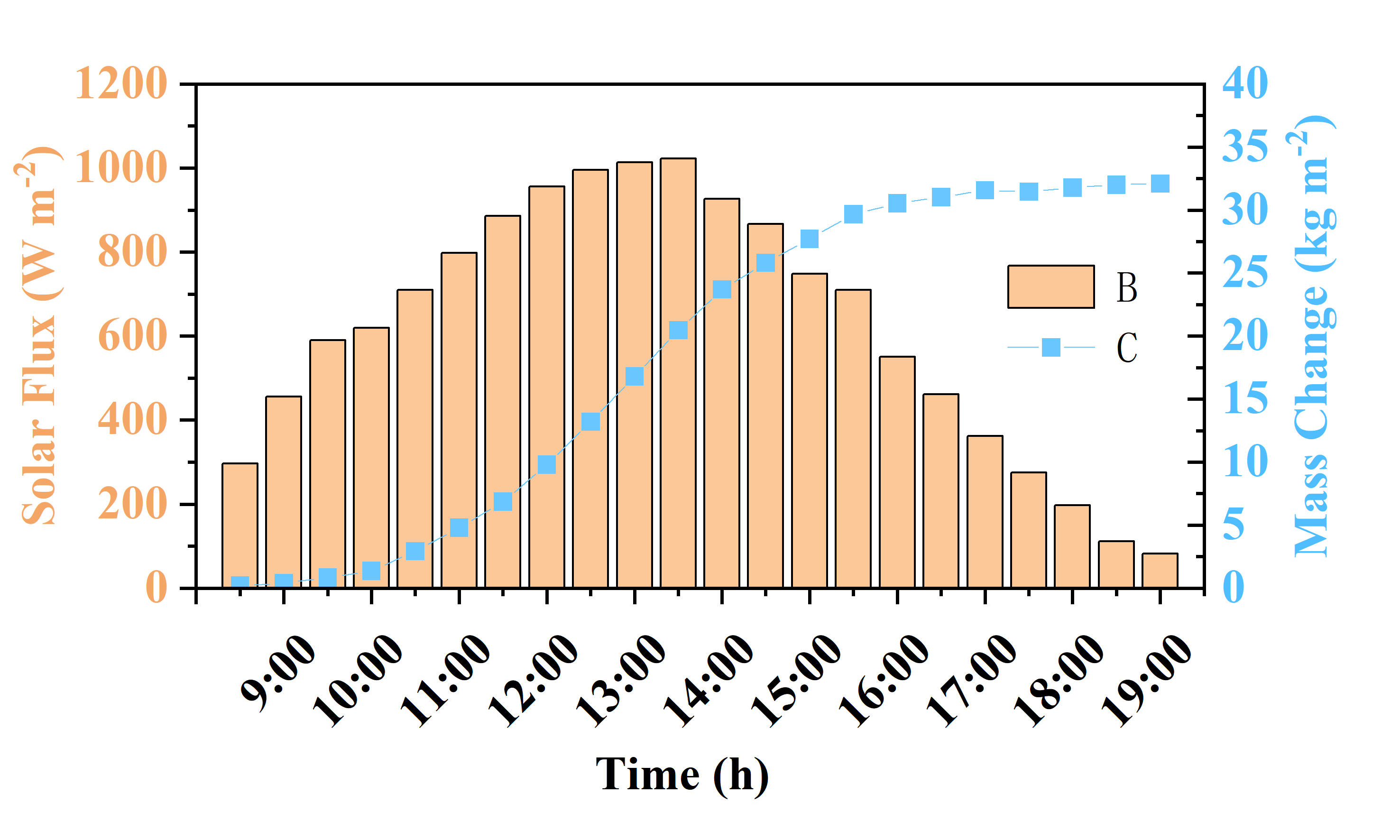
**

**Figure S32.**  The association between the mass change and the intensity of the solar radiation.


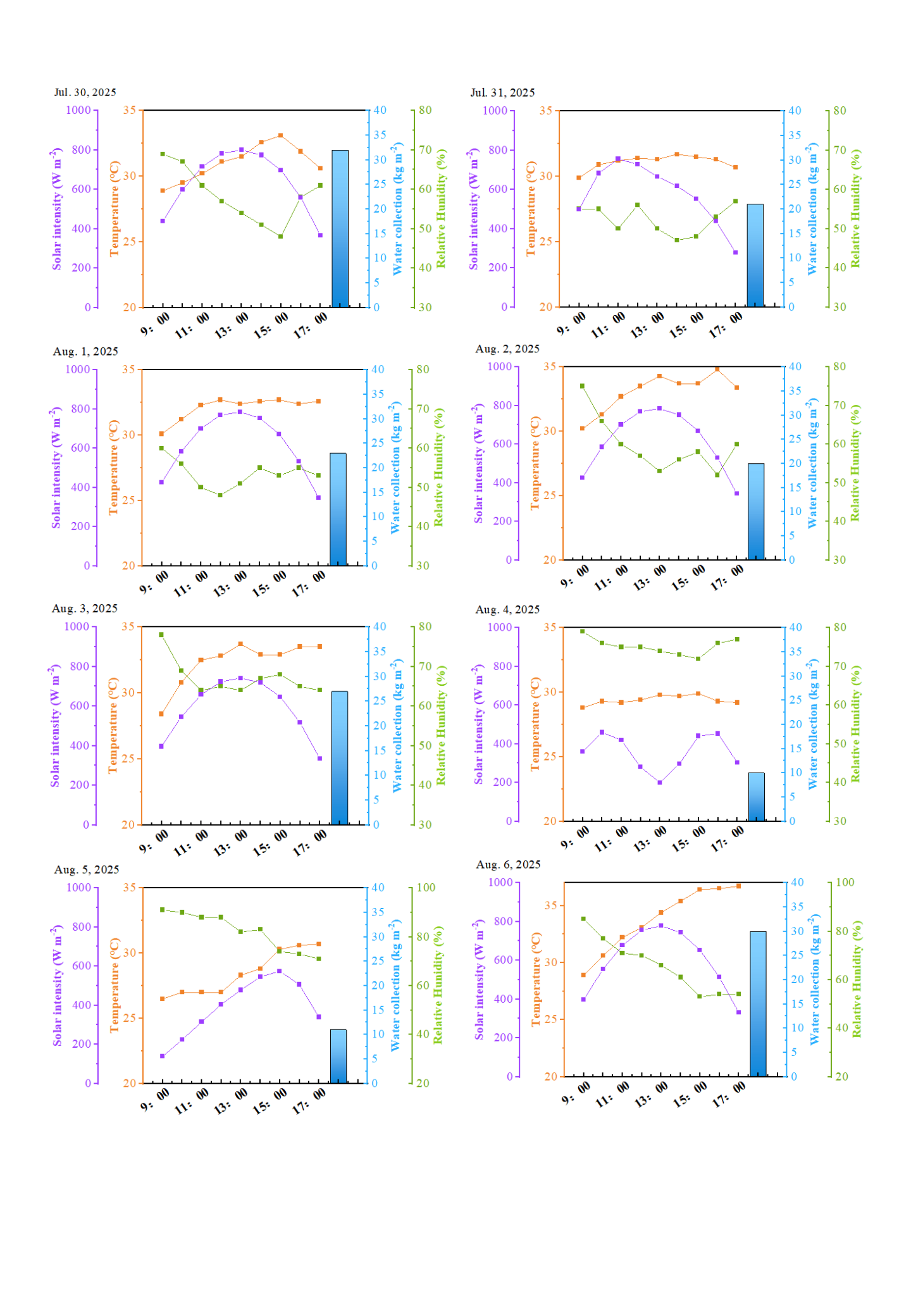


**Figure S33.** The freshwater collection rate and conditions (temperature, solar irradiation and relative humidity) during the outdoor solar desalination tests from July 30 to August 6, 2025.


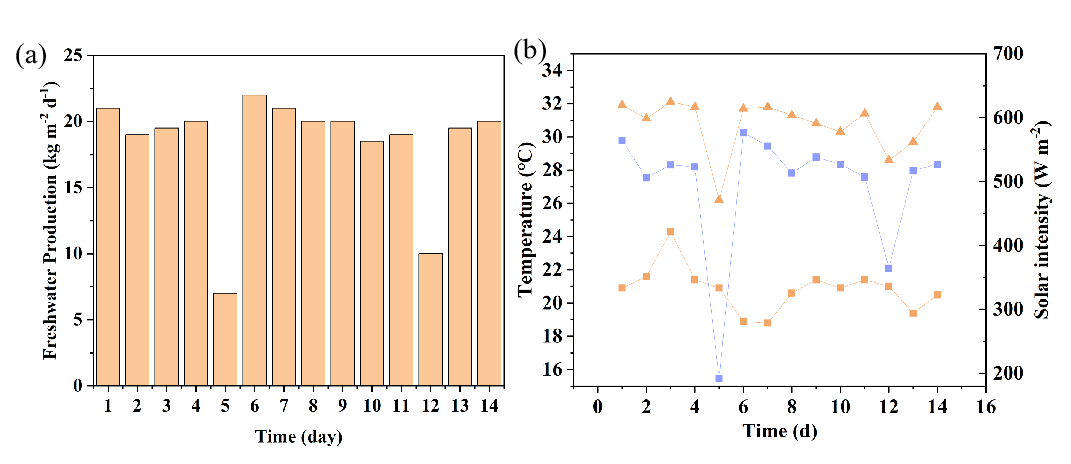


**Figure S34.** The 14-day freshwater production from September 1st to September 14^th^, and the daily conditions during the outdoor testing.


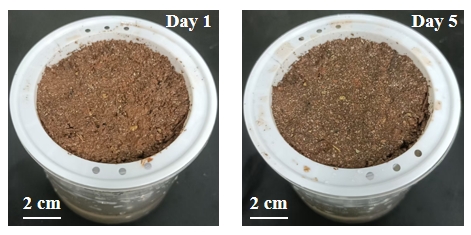


**Figure S35.** Photograph of rice germination situation irrigated by 20 wt.% brine.


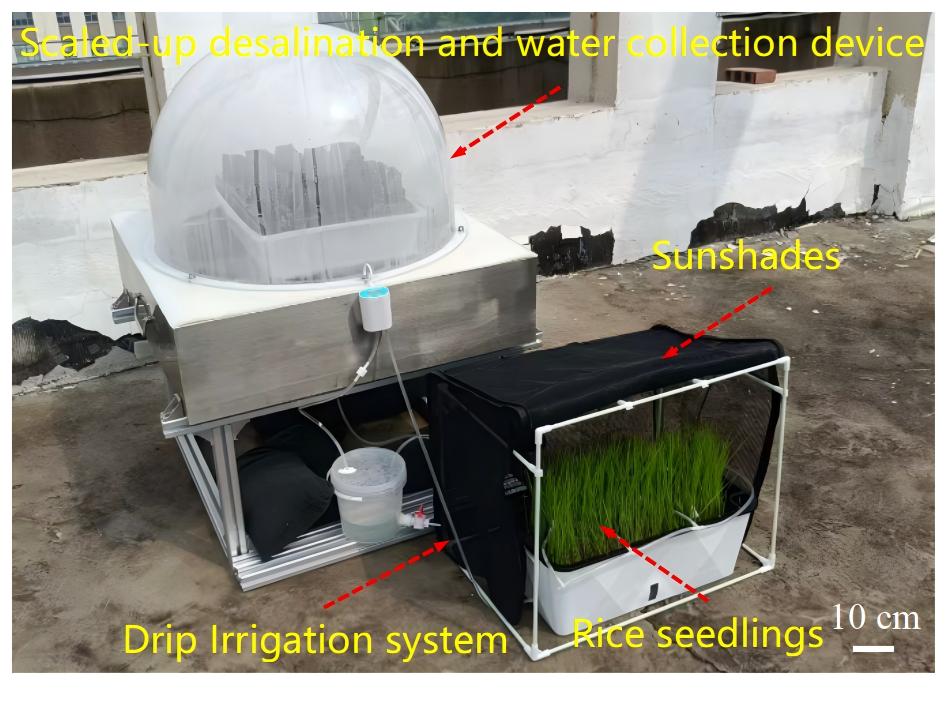


**Figure S36.** Photograph of the scaled-up desalination-irrigation platform.


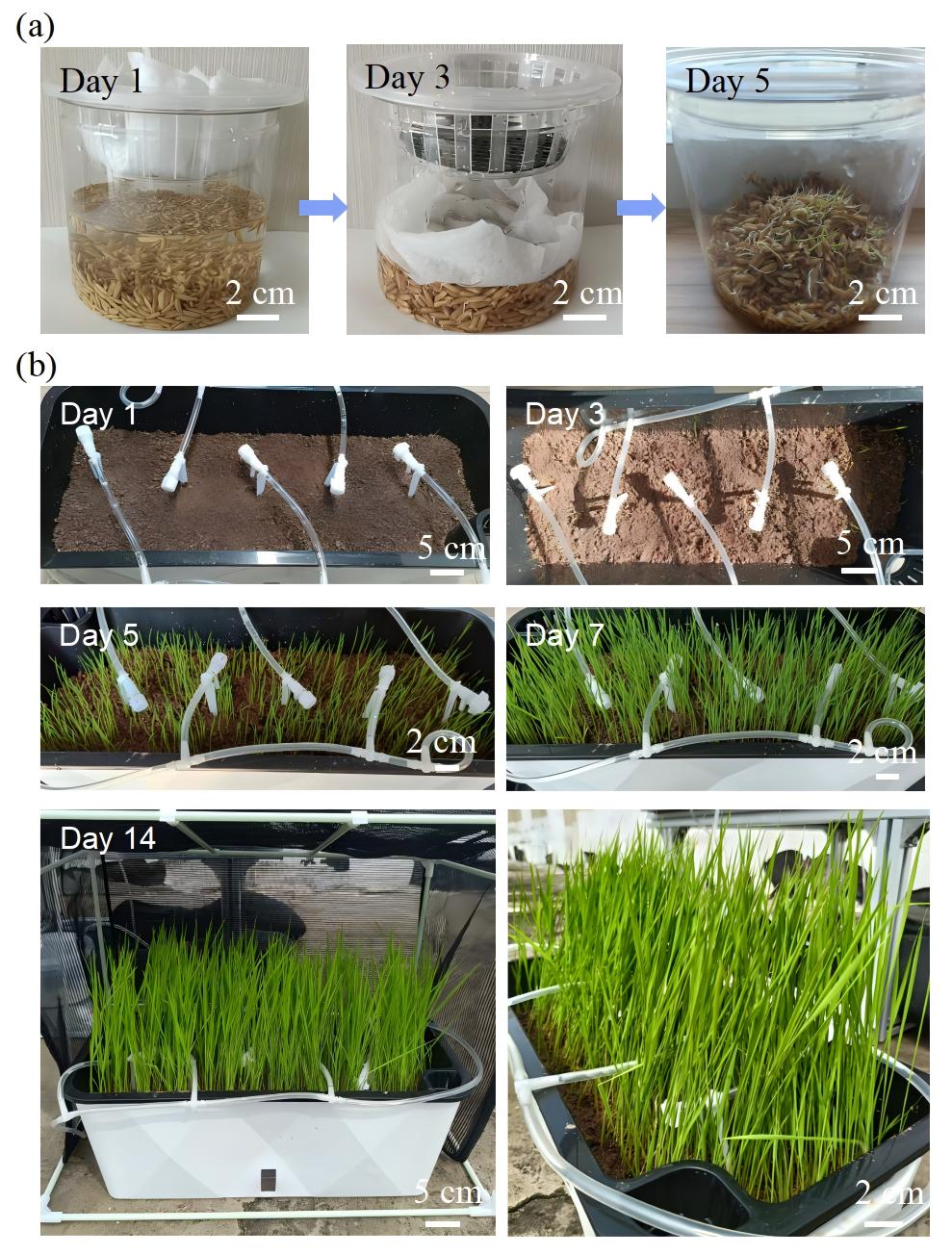


**Figure S37.** Scaled-up germination and cultivation of rice seedlings irrigated with purified water. (a) The germination of rice irrigated with purified water. (b) The growth progression images of rice seedlings irrigated with purified water.

**Supporting Tables**

**Table S1.** The temperature difference between evaporators’ side/top surface and ambient air, with corresponding evaporation rate (ER) .

| Evaporator | *∆T_top_* (℃) | *∆T_side_* (℃) | *ER* (kg m^-2^ h^-1^) | EAI |
| --- | --- | --- | --- | --- |
| Height = 2.4 cm | 1.7 | -1.7 | 3.84 | 5.8 |
| Height = 4 cm | 1.7 | -3.1 | 4.76 | 9 |
| Height = 5 cm | 1.8 | -4.1 | 5.55 | 11 |

Note: *∆T* > 0 represents energy loss to the environment, and *∆T* < 0 represents energy gain from the environment. Notably, The temperature of the top surface is slightly higher than that of the environment, while the temperature of the side surface is much lower than that of the environment, so that the energy obtained by the side surface is greater than the heat loss of the top surface, thus obtaining energy gain and achieving evaporation efficiency beyond the theoretical value. In addition, with the increase of the EAI, the gain of evaporation rate can be further enhanced.

**Table S2.** The salt collection rate of the mycelium-based desalination device compared with those of other previously reported solar evaporators.

| Sample | Salinity (%) | Average evaporation rate  (kg m^-2^ h^-1^) | Salt collection rate  (kg m^-2^ h^-1^) | Ref |
| --- | --- | --- | --- | --- |
| BSE | 25 | 3.98 | 1.27 | [1] |
| CNT/ PDA@PVA | 10 | 2.65 | 0.05916 | [2] |
| SVGC device | 7 | 0.36 | 0.0116 | [3] |
| MPN-OTS | 20 | 2.8 | 0.1 | [4] |
| PVA sponge | 10 | 1.42 | 0.006 | [5] |
| PPy-GF | 10 | 2.58 | 0.152 | [6] |
| Cu-MOF | 3.5 | 1.78 | 0.047 | [7] |
| Janus structure | 10 | 1.21 | 0.037 | [8] |
| TREES | 7 | 2.3 | 0.158 | [9] |
| PV-MS | 17 | 1.17 | 1.02 | [10] |
| ISVG | 3.5 | 2.25 | 0.221 | [11] |
| MSD | 3.5 | 2.21 | 0.06 | [12] |
| D-L-wood | 3.5 | 2.82 | 0.062 | [13] |
| **Evaporator device (Height=5 cm)** | **25** | **8.1±2** | **1.8-1.95** | **This work** |

**Table S3.** The outdoor water production rate of the mycelium-based desalination device compared with those of other previously reported solar evaporation devices under natural sunlight conditions.

| Sample | Average solar intensity (kW m^-2^) | Solution (wt.%) | Water collection rate  (kg m^-2^ h^-1^) | Daily water collection  (kg m^-2^) | Ref |
| --- | --- | --- | --- | --- | --- |
| BSE | 0.797 | Brine, 25 | 3.5 | - | [1] |
| TBEs | 0.84 | Brine, 20 | 4.31 | - | [14] |
| SVGC | 1 | Seawater | - | 2.2 | [3] |
| FDM-2-P-AD | 0.65 | 0 | - | 6.13 (8 h) | [15] |
| LPC-50 mg@CSL | 0.79 | Seawater | 0.985 | 6.025 (10 h) | [16] |
| C/CuSnS FR | 0.54 | Brine, 20 | 1.22 | 4.11 (9h) | [17] |
| CAE | 0.96 | Seawater | - | 16.53 | [18] |
| ISSG | - | Seawater | 3.11 | 24.4 | [19] |
| PGA | - | Seawater | - | 6.8 (10 h) | [20] |
| LC@LCG | 1 | Brine, 3.5 | 1.82 | - | [21] |
| PPCPNA | 0.8 | 0 | 2.78 | 26 | [22] |
| MG | 0.68 | Seawater | - | 7.44 | [23] |
| CNTs-PAAm | - | lake | - | 3.1 | [24] |
| LO-VACFs/CWF | 0.85 | Brine, 3.5 | - | 10.71 | [25] |
| DNH | - | Seawater | 1.52 | - | [26] |
| 3DL Metagel | 0.85 | Seawater | 2.39 | - | [27] |
| RGO/oct | - | Seawater | - | 4.69 | [28] |
| Fe_3_O_4_/V-EVOH | 0.86 | Seawater | - | 7.75 (10 h) | [29] |
| SPP@PAM | 0.85 | Seawater | 1.96 | 9.17 (9 h) | [30] |
| SGA | 0.8 | Seawater | 1.57 | 8.86 | [31] |
| PAAm/QP4VP | - | Seawater | - | 14 | [32] |
| **Evaporator device** | **0.84** | **Seawater** | **1.8-6** | **8-32** | **This work** |

**Table S4.** Calculation of the cost of the evaporator device

| Material | Cost | Remarks |
| --- | --- | --- |
| *Bracket fungi* | ¥ 0 kg^-1^ | The wild *Bracket fungi* is picked from the trees in the wild. |
| Cotton strip | ¥ 1.5 m^-2^ | From JD. com |
| Nylon mesh | ¥ 1.2 m^-2^ | From JD. com |
| Tap-water | 0 | From Hebei University of Technology |
| Mycelium evaporator | ¥ 0 kg^-1^ | The mycelium can be prepared by cutting down the hard wood-like shell of the Bracket fungi. |
| Evaporator device | ¥ 1.325 m^-3^ | Each evaporator requires 36 cm^-2^ nylon mesh, and 3 cm^-2^ cotton strip. |

**Supporting References**

1. Y. Z. Li, J. Zhang, D. Yao, et al., “Wetting‐Induced Blackening Enables High‐Efficiency Solar Evaporation in Fully Biomaterial‐Based Porous Hydrogels Without Photothermal Additives,” Advanced Functional Materials, (2025), e19930.

https://doi.org/10.1002/adfm.202519930

2. M. Ding, D. Zhao, Z. Duan, et al., “Bio-inspired Solar Evaporators for Stable and Efficient Desalination of High-salinity Brine with Zero Liquid Discharge,” *Science Bulletin* (2025).

https://doi.org/10.1016/j.scib.2025.04.071

3. Y. Xu, Y. Tang, L. Liu, et al., “Hofmeister effect-based 3D hydrogel sponge via non-contact localized crystallization for achieving zero liquid discharge desalination of high-salinity brine,” *Desalination* 610, (2025): 118911.

https://doi.org/10.1016/j.desal.2025.118911

4. M. A. Abdelsalam, M. Sajjad, A. Raza, F. AlMarzooqi, and T. Zhang, “Sustainable Biomimetic Solar Distillation with Edge Crystallization for Passive Salt Collection and Zero Brine Discharge,” *Nature Communications* 15, 1 (2024): 874.

https://doi.org/10.1038/s41467-024-45108-2

5. Y. Zhang, Q. Zhong, Q. Huang, et al., “Surface Engineering of 3D Solar Evaporator for Uncompromising Water Evaporation and Salt Production Toward High Concentration Brine,” *Advanced Functional Materials* 34, 48 (2024): 2408554.

https://doi.org/10.1002/adfm.202408554

6. T. X. Zhang, J. X. Zhao, L. Liang, and C. L. Guo, “Constructing a Solar Evaporator with Salt-Collecting Paper by Stacking Hydrophilic Sponges for Freshwater Production and Salt Collection,” *Acs Applied Materials & Interfaces* 14, 1 (2022): 668.

https://doi.org/10.1021/acsami.1c17534

7. Z. Yu, S. N. Li, Y. Chen, et al., “Intensifying the co-production of vapor and salts by a one-way brine-flowing structure driven by solar irradiation or waste heat,” *Desalination* 539, (2022): 115942.

https://doi.org/10.1016/j.desal.2022.115942

8. X. Ma, X. Y. Wan, Z. Fang, et al., “Orientational seawater transportation through Cu(TCNQ) nanorod arrays for efficient solar desalination and salt production,” *Desalination* 522, (2022): 115399.

https://doi.org/10.1016/j.desal.2021.115399

9. R. A. Gu, Z. Yu, Y. Sun, et al., “Enhancing stability of interfacial solar evaporator in high-salinity solutions by managing salt precipitation with Janus-based directional salt transfer structure,” *Desalination* 524, (2022): 115470.

https://doi.org/10.1016/j.desal.2021.115470

10. Y. Bian, Z. H. Ye, G. Y. Zhao, et al., “Enhanced Contactless Salt-Collecting Solar Desalination,” *Acs Applied Materials & Interfaces* 14, 29 (2022): 34151.

https://doi.org/10.1021/acsami.2c09063

11. L. P. Yang, T. Y. Sun, J. B. Tang, et al., “Photovoltaic-multistage desalination of hypersaline waters for simultaneous electricity, water and salt harvesting via automatic rinsing,” *Nano Energy* 87, (2021): 106163.

https://doi.org/10.1016/j.nanoen.2021.106163

12. Y. Tian, Y. Jiang, R. Zhu, et al., “Solar-Driven Multistage Device Integrating Dropwise Condensation and Guided Water Transport for Efficient Freshwater and Salt Collection,” *Environmental Science & Technology* 58, 17 (2024): 7335.

https://doi.org/10.1021/acs.est.3c10450

13. Y. Li, S. Cheng, Z. Yu, R. Gu, and X. He, “Stable, zero liquid discharge, and highly efficient solar-driven multistage distillation device based on tree-inspired radial water transfer,” *Journal of Cleaner Production* 375, (2022): 134025.

https://doi.org/10.1016/j.jclepro.2022.134025

14. Y. Chen, R. Hou, L. Yang, et al., “Elastic, Janus 3D evaporator with arch-shaped design for low-footprint and high-performance solar-driven zero-liquid discharge,” *Desalination* 583, (2024): 117644.

https://doi.org/10.1016/j.desal.2024.117644

15. M. Ding, Z. Duan, D. Zhao, C. Y. Liu, and C. Li, “Tree‐Inspired 3D Biomimetic Evaporator for Efficient Solar Desalination and Automated Salt Collection with Zero Liquid Discharge,” *Advanced Functional Materials* 10.1002/adfm.202512220, (2025): e12220.

https://doi.org/10.1002/adfm.202512220

16. Y. Cao, J. Wang, W. Guan, et al., “Spatially regulated water-heat transport by fluidic diode membrane for efficient solar-powered desalination and electricity generation,” *Nature Communications* 16, 1 (2025): 5050.

https://doi.org/10.1038/s41467-025-60283-6

17. S. Chen, B. Yang, D. Yang, X. Qiu, and D. Zheng, “Multifunctional Fully Biomass-Derived Bilayer Aerogel for Efficient Solar-Driven Desalination and Thermoelectricity Generation,” *ACS Nano* 19, 21 (2025): 19681.

https://doi.org/10.1021/acsnano.5c01360

18. H. Jiang, X. Liu, H. Wang, et al., “Waterwheel-inspired rotating evaporator for efficient and stable solar desalination even in saturated brine,” *Science Bulletin* 68, 15 (2023): 1640.

https://doi.org/10.1016/j.scib.2023.07.011

19. W. Li, J. Guan, T. Hao, et al., “Collapsible and portable solar-driven directional backside evaporation–condensation system for highly efficient freshwater generation and collection,” *Chemical Engineering Journal* 518, (2025): 164750.

https://doi.org/10.1016/j.cej.2025.164750

20. W. Li, J. Li, L. Ding, et al., “Interfacial Assembled Hydrogel Evaporator for Highly Efficient Thermal Management and Photothermal Coupled Water Splitting Reaction,” *Advanced Functional Materials* 34, 52 (2024): 2411387.

https://doi.org/10.1002/adfm.202411387

21. Z. Li, Y. Wang, Q. Huang, et al., “Plant-inspired gradient-pore aerogel achieving both fast water transport and low evaporation enthalpy for interfacial evaporation,” *Chemical Engineering Journal* 519, (2025): 165499.

https://doi.org/10.1016/j.cej.2025.165499

22. X. Lin, P. Wang, R. Hong, et al., “Fully Lignocellulosic Biomass‐Based Double‐Layered Porous Hydrogel for Efficient Solar Steam Generation,” *Advanced Functional Materials* 32, 51 (2022): 2209262.

https://doi.org/10.1002/adfm.202209262

23. W. Ma, T. Lu, W. Cao, R. Xiong, and C. Huang, “Bioinspired Nanofibrous Aerogel with Vertically Aligned Channels for Efficient Water Purification and Salt‐Rejecting Solar Desalination,” *Advanced Functional Materials* 33, 23 (2023): 2214157.

https://doi.org/10.1002/adfm.202214157

24. Z. Mao, Y. Han, J. Shen, et al., “Simultaneous Salt Rejection and Heat Localization Via Engineering Macrochannels in Morning Glory‐Shaped 3D Evaporator,” *Advanced Science* 11, 40 (2024): 2405639.

https://doi.org/10.1002/advs.202405639

25. J. Qiu, X. Xu, Z. Li, et al., “A Solar-electric Dual-driven Microporous Hydrogel Evaporator for All-weather Highly Efficient Water Purification,” *Nano Energy* 130, (2024): 110057.

https://doi.org/10.1016/j.nanoen.2024.110057

26. Y. Tian, R. Song, Y. Li, et al., “Biomimetic Structural Design of Fabric for Low-Cost, Scalable, and Highly Efficient Off-Grid Solar-Driven Water Purification,” *Advanced Functional Materials* 34, 19 (2024): 2309470.

https://doi.org/10.1002/adfm.202309470

27. B. Wang, H. Cheng, H. Zhu, and L. Qu, “Hierarchically Structured Hydrogels for Rapid Solar Vapor Generation with Super Resistance to Salt,” *Advanced Functional Materials* 35, 28 (2025): 2500459.

https://doi.org/10.1002/adfm.202500459

28. D. Wang, R. Zhu, X. Tang, et al., “Multi-bionic Strategies Integration in Cellulose Nanofiber-Based Metagels with Strong Hydrogen-Bonded Network for Solar-Driven Water Evaporation,” *Advanced Fiber Materials* 7, 3 (2025): 748.

https://doi.org/10.1007/s42765-025-00517-w

29. J. Wu, P. Min, G. Yin, Z. Z. Yu, and X. Li, “Three‐Phase Emulsion Derived Solar Thermal Reduced Graphene Oxide/Octadecane Phase Change Foam for Salt Resistant Day-Night Water Evaporation,” *Advanced Functional Materials* (2025): 2501541.

https://doi.org/10.1002/adfm.202501541

30. Y. Wu, S. Li, K. Yan, et al., “Biomimetic Design of 3D Fe3O4/V-EVOH Fiber-Based Self-Floating Composite Aerogel to Enhance Solar Steam Generation Performance,” *Nano Letters* 24, 15 (2024): 4537.

https://doi.org/10.1021/acs.nanolett.4c00572

31. G. Yang, Z. Yin, X. Han, et al., “Pyramidal array Janus hydrogel-based solar evaporator via broadband light trapping inspired by durian peel for efficient seawater desalination,” *Water Research* 287, (2025): 124375.

https://doi.org/10.1016/j.watres.2025.124375

32. X. Zhao, H. Zhang, K.-Y. Chan, et al., “Tree-Inspired Structurally Graded Aerogel with Synergistic Water, Salt, and Thermal Transport for High-Salinity Solar-Powered Evaporation,” *Nano-Micro Letters* 16, 1 (2024): 222.

https://doi.org/10.1007/s40820-024-01448-8

33. J. Zhu, S. Qiu, M. Duan, et al., “Polyelectrolyte Gradient Hydrogels for Efficient Solar Evaporation,” *Advanced Functional Materials* 10.1002/adfm.202512350, (2025): e12350.

https://doi.org/10.1002/adfm.202512350
